# Supplementary material for: Oral pre-exposure prophylaxis retention among men who have sex with men and transgender persons: Systematic review and meta-analysis
Source: PLoS One. 2025 Oct 17;20(10):e0333494. doi: 10.1371/journal.pone.0333494 (PMC12533894; doi:10.1371/journal.pone.0333494)
Supplement: S1 Table — (DOCX) [file pone.0333494.s001.docx]

**S1 Table. Search strategy.**

| **Search strategy** |  |
| --- | --- |
| PubMed | (("PrEP") OR ("pre-exposure prophylaxis") OR ("preexposure prophylaxis") OR ("Pre-Exposure Prophylaxis"[MeSH])) AND (("HIV") OR ("HIV Infections/prevention and control"[MAJR])) AND (("men who have sex with men") OR ("transgender") OR ("bisexual") OR ("gay") OR ("Transgender Persons"[MeSH]) OR ("Homosexuality, Male"[MeSH]) OR ("Bisexuality"[MeSH])) |
| Ovid | (("PrEP") OR ("pre-exposure prophylaxis") OR ("preexposure prophylaxis")) AND (("HIV") OR ("human immunodeficiency virus")) AND (("men who have sex with men") OR (“homosexuality”) OR ("gay") OR ("bisexual") OR ("transgender")) |
