# Supplementary material for: Oral pre-exposure prophylaxis retention among men who have sex with men and transgender persons: Systematic review and meta-analysis
Source: PLoS One. 2025 Oct 17;20(10):e0333494. doi: 10.1371/journal.pone.0333494 (PMC12533894; doi:10.1371/journal.pone.0333494)
Supplement: S2 Table — This table also includes the citations for every study; see S1 References for reference list. †We evaluated comparability on the basis of the analysis and not the design (as studies were single-arm). This evaluation was carried out as follows: whether proportions were adjusted for age and (if diverse populations were included) gender (one star), and whether proportions were additionally adjusted for other factors (one star). The comparability criteria were applied similarly for randomized control trials and observational studies. ‡Randomized control trials were included in the systematic review as separate cohort studies and thus the Newcastle-Ottawa Quality Assessment Scale for cohort studies was applied hereto. (DOCX) [file pone.0333494.s002.docx]

**S2 Table. Risk of bias assessment using the Newcastle-Ottawa Scale (NOS) for assessing the quality of nonrandomized studies in this systematic review and meta-analysis.**

| **Study**  *(Author, year)* | **Selection**  Maximum score: **** | **Comparability**†  Maximum score: ** | **Outcome**  Maximum score: *** | **Total score**  Maximum score: *9* | **Appraisal** |
| --- | --- | --- | --- | --- | --- |
| **Ahaus, 2020 [1]** | **** |  | *** | 7 | Included |
| **Akbar, 2020 [2]** | *** |  | *** | 6 | Included |
| **Akolo, 2020**‡ **[3]** | **** | * | ** | 7 | Included |
| **Bhatia, 2021 [4]** | *** |  | *** | 6 | Included |
| **Blaylock, 2018 [5]** | *** | * | *** | 7 | Included |
| **Blumenthal, 2017 [6]** | ** | * | ** | 5 | Included |
| **Chan, 2016 [7]** | **** | * | *** | 8 | Included |
| **Chan, 2019 [8]** | **** | ** | *** | 9 | Included |
| **Chinbunchorn, 2020 [9]** | *** | * | ** | 6 | Included |
| **Clement, 2019 [10]** | *** |  | ** | 5 | Included |
| **Colson, 2020 [11]** | **** | * | *** | 8 | Included |
| **Coyer, 2020 [12]** | **** | ** | ** | 8 | Included |
| **Doblecki-Lewis, 2018 [13]** | **** | ** | ** | 8 | Included |
| **Dourado, 2021 [14]** | *** |  | ** | 5 | Included |
| **Edelman, 2017 [15]** | *** |  | *** | 6 | Included |
| **Egan, 2020 [16]** | **** | * | *** | 8 | Included |
| **Georgescu, 2017 [17]** | **** |  | ** | 6 | Included |
| **Golub, 2018 [18]** | *** |  | ** | 5 | Included |
| **Grant, 2014 [19]** | *** | ** | ** | 7 | Included |
| **Grant, 2018**‡ **[20]** | **** | * | **** | 9 | Included |
| **Greenwald, 2018 [21]** | **** | * | *** | 8 | Included |
| **Grinsztejn, 2018 [22]** | **** | ** | *** | 9 | Included |
| **Grulich, 2018 [23]** | **** |  | *** | 7 | Included |
| **Havens, 2019 [24]** | **** |  | *** | 7 | Included |
| **Hickey, 2020 [25]** | *** |  | ** | 5 | Included |
| **Hoenigl, 2018**‡ **[26]** | **** | ** | *** | 9 | Included |
| **Hojilla, 2018 [27]** | *** | * | *** | 7 | Included |
| **Hosek, 2017 [28]** | **** | ** | *** | 9 | Included |
| **Hoth, 2019 [29]** | **** |  | *** | 7 | Included |
| **Huang, 2019 [30]** | **** | ** | *** | 9 | Included |
| **Hucks-Ortiz, 2016 [31]** | *** |  | *** | 6 | Included |
| **Iniesta, 2021 [32]** | *** | ** | *** | 8 | Included |
| **Kaewpoowat, 2019 [33]** | *** |  | *** | 6 | Included |
| **Kimani, 2021 [34]** | **** |  | *** | 7 | Included |
| **Kyongo, 2018 [35]** | **** |  | *** | 7 | Included |
| **Lal, 2017 [36]** | **** | * | *** | 8 | Included |
| **Lalley-Chareczko, 2017 [37]** | **** |  | *** | 7 | Included |
| **Landovitz, 2017 [38]** | **** | * | *** | 8 | Included |
| **Lee, 2019 [39]** | *** |  | ** | 5 | Included |
| **Liu, 2015 [40]** | **** |  | *** | 7 | Included |
| **Liu, 2016 [41]** | **** | ** | *** | 9 | Included |
| **Liu, 2019**‡ **[42]** | *** | ** | *** | 8 | Included |
| **Marins, 2019 [43]** | **** |  | *** | 7 | Included |
| **McAllister, 2019 [44]** | **** |  | *** | 7 | Included |
| **Medland, 2020 [45]** | **** |  | *** | 7 | Included |
| **Mehrotra, 2021 [46]** | *** |  | ** | 5 | Included |
| **Mehta, 2020 [47]** | **** | * | ** | 7 | Included |
| **Milam, 2019 [48]** | *** | ** | *** | 8 | Included |
| **Miltz, 2019**‡ **[49]** | *** | ** | ** | 7 | Included |
| **Molina, 2017 [50]** | **** |  | **** | 8 | Included |
| **Montano, 2018 [51]** | **** | ** | *** | 9 | Included |
| **Montgomery, 2016 [52]** | **** |  | *** | 7 | Included |
| **Moore, 2018**‡ **[53]** | **** | ** | *** | 9 | Included |
| **Newcomb, 2019 [54]** | *** | ** | *** | 8 | Included |
| **Nguyen, 2018 [55]** | *** | ** | *** | 8 | Included |
| **Nostlinger, 2020 [56]** | *** |  | ** | 5 | Included |
| **Page, 2018 [57]** | **** |  | *** | 7 | Included |
| **Parisi, 2018 [58]** | **** |  | *** | 7 | Included |
| **Paulino-Ramirez, 2019 [59]** | **** |  | *** | 7 | Included |
| **Pornpaisalsakul, 2020**‡ **[60]** | **** |  | *** | 7 | Included |
| **Reback, 2018 [61]** | *** |  | ** | 5 | Included |
| **Reback, 2019 [62]** | **** |  | *** | 7 | Included |
| **Refugio, 2019 [63]** | **** |  | ** | 6 | Included |
| **Rusie, 2018 [64]** | **** | * | *** | 8 | Included |
| **Schumacher, 2020 [65]** | **** | ** | *** | 9 | Included |
| **Selfridge, 2020 [66]** | **** | * | *** | 8 | Included |
| **Serota, 2020 [67]** | **** | * | *** | 8 | Included |
| **Shover, 2018 [68]** | **** |  | *** | 7 | Included |
| **Songtaweesin, 2020**‡ **[69]** | **** | * | *** | 8 | Included |
| **Songtaweesin, 2020 [70]** | **** |  | *** | 7 | Included |
| **Spinelli, 2019 [71]** | **** | ** | *** | 9 | Included |
| **Stekler, 2018 [72]** | **** |  | ** | 6 | Included |
| **Tan, 2018 [73]** | **** | * | *** | 8 | Included |
| **Tung, 2017 [74]** | *** |  | *** | 6 | Included |
| **Vaccher, 2019 [75]** | **** |  | *** | 7 | Included |
| **Veloso, 2019 [76]** | **** |  | *** | 7 | Included |
| **Veloso, 2020 [77]** | *** |  | *** | 6 | Included |
| **Volk, 2020 [78]** | **** |  | *** | 7 | Included |
| **Wheeler, 2016 [79]** | *** |  | ** | 5 | Included |
| **Wheeler, 2019 [80]** | **** | * | *** | 8 | Included |
| **Wirtz, 2020 [81]** | **** | ** | *** | 9 | Included |
| **Wu, 2019 [82]** | **** |  | *** | 7 | Included |
| **Wu, 2020 [83]** | *** | ** | *** | 8 | Included |
| **Zablotska, 2018 [84]** | **** |  | *** | 7 | Included |

This table also includes the citations for every study; see S1 References for reference list.

†We evaluated comparability on the basis of the analysis and not the design (as studies were single-arm). This evaluation was carried out as follows: whether proportions were adjusted for age and (if diverse populations were included) gender (one star), and whether proportions were additionally adjusted for other factors (one star). The comparability criteria were applied similarly for randomized control trials and observational studies.

‡Randomized control trials were included in the systematic review as separate cohort studies and thus the Newcastle-Ottawa Quality Assessment Scale for cohort studies was applied hereto.
