# Supplementary material for: Oral pre-exposure prophylaxis retention among men who have sex with men and transgender persons: Systematic review and meta-analysis
Source: PLoS One. 2025 Oct 17;20(10):e0333494. doi: 10.1371/journal.pone.0333494 (PMC12533894; doi:10.1371/journal.pone.0333494)
Supplement: S3 Table — Abbreviations: MSM = men who have sex with men; TGP = transgender person; PrEP = pre-exposure prophylaxis; IQR = interquartile range; N.A. = not applicable because missing or not reported. (DOCX) [file pone.0333494.s003.docx]

**S3 Table. Characteristics of included studies.**

| **Study**  *(Author, year)* | **Design** | **Duration** | **Country** | **Sample size** | **Target population** | **Percentage MSM** | **PrEP regimen** | **Age**  *Median (IQR)* | **Ethnicity** | **PrEP financing** |
| --- | --- | --- | --- | --- | --- | --- | --- | --- | --- | --- |
| **Ahaus, 2020** | Prospective cohort study | 2017-2018 | DEU | 139 | MSM | 98.6 | Both | 38 | N.A. | Partial contribution |
| **Akbar, 2020** | Demonstration study | 2018-2018 | MYS | 150 | MSM | 100 | Daily | N.A. | Mixed | Free of charge |
| **Akolo, (intervention arm) 2020** | RCT | N.A. | KEN | 84 | MSM | 100 | N.A. | N.A. | N.A. | N.A. |
| **Akolo, (standard care arm) 2020** | RCT | N.A. | KEN | 84 | MSM | 100 | N.A. | N.A. | N.A. | N.A. |
| **Bhatia, 2021** | Implementation study | 2019-2020 | VNM | 1002 | MSM | 96 | N.A. | N.A. | N.A. | N.A. |
| **Blaylock, 2018** | Cohort study | 2013-2016 | USA | 159 | MSM | 98 | Mixed | N.A. | N.A. | N.A. |
| **Blumenthal, 2017** | Demonstration study | N.A. | USA | 398 | MSM | 100 | N.A. | N.A. | N.A. | N.A. |
| **Chan, 2016** | Prospective cohort study | 2014-2015 | USA | 139 | MSM, TGP and women | n.a. | Daily | N.A. | Mixed | Free of charge |
| **Chan, 2019** | Implementation study | 2013-2015 | USA | 282 | MSM | 99 | Daily | N.A. | Mixed | N.A. |
| **Chinbunchorn, 2020** | Implementation study | 2016-2019 | THA | 5687 | MSM and TGP | 80 | N.A. | N.A. | N.A. | Prep-15: fee, Princess prep: free |
| **Clement, 2019** | Retrospective cohort study | 2015-2018 | USA | 84 | MSM | 82 | N.A. | 27 | Mixed | Free of charge |
| **Colson, (intervention arm) 2020** | RCT in observational cohort | 2015-2017 | USA | 101 | MSM | 94 | N.A. | 31 | Black | N.A. |
| **Colson, (standard arm) 2020** | RCT in observational cohort | 2015-2017 | USA | 103 | MSM | 96 | N.A. | 31 | Black | N.A. |
| **Coyer, 2020** | Demonstration study | 2015-2019 | NLD | 367 | MSM | 99.5 | Both | 40.4 (32.6-48.5) | Mixed | Free of charge |
| **Doblecki-Lewis, 2018** | Demonstration study | 2012-2014 | USA | 554 | MSM | N.A. | Daily | 33 | Mixed | Free of charge |
| **Dourado, 2021** | Demonstration study | 2019-2020 | BRA | 538 | MSM and TGP | N.A. | N.A. | N.A. | N.A. | N.A. |
| **Edelman, 2017** | Observational cohort study | 2015-2016 | USA | 131 | MSM | 100 | Daily | N.A. | Mixed | N.A. |
| **Egan, 2020** | Observational cohort study | 2016-2017 | USA | 54 | MSM | 100 | Daily | N.A. | Mixed | Free of charge |
| **Georgescu, 2017** | Retrospective cohort study | 2014-2016 | USA | 65 | MSM | 91 | N.A. | N.A. | Mixed | N.A. |
| **Golub, 2018** | Demonstration/ implementation study | N.A. | USA | 300 | MSM and TGP | N.A. | N.A. | N.A. | N.A. | N.A. |
| **Grant, 2014** | Open label extension study | 2011-2012 | Multiple | 1128 | MSM and TGP | N.A. | Daily | N.A. | Mixed | Free of charge |
| **Grant, 2018** | Randomized open label study | 2012-2014 | THA and USA | 357 | MSM | 98 | Both | N.A. | Mixed | N.A. |
| **Greenwald, 2018** | Retrospective cohort study | 2011-2018 | CAN | 1473 | MSM | 98 | Both | 36 (29-45) | Mixed | N.A. |
| **Grinsztejn, 2018** | Demonstration study | 2014-2016 | BRA | 450 | MSM | 94.4 | Daily | N.A. | Mixed | Free of charge |
| **Grulich, 2018** | Implementation study | 2016-2017 | AUS | 3700 | MSM | 99 | Daily | 36 (30-45) | Mixed | Free of charge |
| **Havens, 2019** | Pilot study (prospective observational cohort) | 2017-2017 | USA | 60 | MSM | 91.7 | N.A. | N.A. | Mixed | Free of charge |
| **Hickey, 2020** | Open label extension study | 2020-2020 | THA | 590 | MSM | 100 | Daily | N.A. | Asian | N.A. |
| **Hoenigl, 2018** | RCT | 2014-2016 | USA | 394 | MSM | 99 | Daily | 33 (28-41) | Mixed | N.A. |
| **Hojilla, 2018** | Retrospective cohort study | 2014-2015 | USA | 344 | MSM | 100 | N.A. | 31 | Mixed | Dependent on insurance |
| **Hosek, 2017** | Demonstration study | 2013-2014 | USA | 72 | MSM | 100 | Daily | N.A. | Mixed | N.A. |
| **Hoth, 2019** | Implementation study | 2017-2018 | USA | 127 | MSM | 88 | N.A. | 32 | Mixed | Dependent on insurance |
| **Huang, 2019** | Retrospective cohort study | 2012-2016 | USA | 7250 | MSM | 98.2 | Daily | N.A. | N.A. | N.A. |
| **Hucks-Ortiz, 2016** | Prospective cohort study | 2016-2016 | USA | 226 | MSM | 100 | Daily | N.A. | Black | N.A. |
| **Iniesta, 2021** | Prospective observational cohort study | 2017-2019 | ESP | 321 | MSM | 99.1 | Daily | 36 (31-42) | Mixed | Free of charge |
| **Kaewpoowat, 2019** | Demonstration study | 2015-2017 | THA | 60 | MSM | 82 | Daily | 21 | Asian | N.A. |
| **Kimani, 2021** | Prospective cohort study | 2018-2018 | KEN | 53 | MSM and TGP | 79 | Daily | N.A. | N.A. | Free of charge |
| **Kyongo, 2018** | Demonstration study | 2015-2016 | KEN | 438 | MSM | 100 | N.A. | N.A. | N.A. | N.A. |
| **Lal, 2017** | Demonstration study | 2014-2015 | AUS | 106 | MSM | 99 | Daily | 34 (30.8-45) | Mixed | Full price charge |
| **Lalley-Chareczko, 2017** | Prospective observational cohort | N.A. | USA | 50 | MSM | 90 | N.A. | 22.4 | Mixed | N.A. |
| **Landovitz, 2017** | Open-label single arm interventional cohort study | 2014-2016 | USA | 300 | MSM | 99.7 | Daily | 34 | Mixed | N.A. |
| **Lee, 2019** | Pilot study (prospective observational cohort) | 2017-2018 | CHN | 71 | MSM | 100 | Daily | 32 (27-40) | Asian | Partial contribution |
| **Liu, 2019**  (Intervention arm) | RCT intervention in observational cohort study | 2015-2016 | USA | 81 | MSM | 96 | Daily | N.A. | Mixed | Free of charge |
| **Liu, 2019**  (Standard of care arm) | RCT intervention in observational cohort study | 2015-2016 | USA | 40 | MSM | 93 | Daily | N.A. | Mixed | Free of charge |
| **Liu, 2015** | Demonstration study | 2012-2014 | USA | 557 | MSM and TGP | N.A. | Daily | N.A. | N.A. | N.A. |
| **Liu, 2016** | Demonstration study | 2012-2015 | USA | 557 | MSM | 98.4 | Daily | N.A. | Mixed | Free of charge |
| **Marins, 2019** | Prospective, multicenter, open-label demonstration project | 2014-2016 | BRA | 450 | MSM | 94.4 | Daily | N.A. | Mixed | Free of charge |
| **McAllister, 2019** | Demonstration study | N.A. | AUS | 185 | MSM | 100 | Daily | N.A. | N.A. | N.A. |
| **Medland, 2020** | N.A. | 2018-2019 | AUS | 29,618 | MSM | 98.7 | Daily | 35 (28-45) | N.A. | N.A. |
| **Mehrotra, 2021** | Observational cohort study | 2017-2019 | USA | 1092 | MSM | 97.2 | N.A. | N.A. | Mixed | N.A. |
| **Mehta, 2020** | Observational cohort study | 2017-2019 | KEN | 158 | MSM | 100 | N.A. | 24 | N.A. | N.A. |
| **Milam, 2019** | Demonstration study | 2013-2015 | USA | 398 | MSM | 99.3 | N.A. | N.A. | Mixed | Free of charge |
| **Miltz, 2019** | Open label randomized trial | 2012-2016 | GBR | 540 | MSM | 99.4 | Daily | 35 (29-42) | Mixed | Free of charge |
| **Molina, 2017** | Open label extension study of the ANRS IPERGAY trial | 2014-2015 | Multiple | 361 | MSM | 99 | Event-driven | 37 (30-44) | Mixed | N.A. |
| **Montano, 2018** | Observational cohort study | 2014-2017 | USA | 183 | MSM | 100 | N.A. | N.A. | Mixed | N.A. |
| **Montgomery, 2016** | Observational cohort study | 2013-2014 | USA | 50 | MSM | 100 | Daily | N.A. | Mixed | N.A. |
| **Moore, 2018**  (Intervention arm) | RCT | 2013-2016 | USA | 200 | MSM | 98.5 | Daily | N.A. | Mixed | N.A. |
| **Moore, 2018**  (Standard of care arm) | RCT | 2013-2016 | USA | 198 | MSM | 100 | Daily | N.A. | Mixed | N.A. |
| **Newcomb, 2019** | Observational cohort study | 2015-2018 | USA | 1100 | MSM and TGP | N.A. | N.A. | N.A. | N.A. | N.A. |
| **Nguyen, 2018** | Observational cohort study | 2010-2015 | CAN | 109 | MSM | 100 | Daily | 36 (31-44) | Mixed | N.A. |
| **Nostlinger, 2020** | Observational cohort study | 2015-2017 | BEL | 200 | MSM | 98.5 | Both | 38 (22-70) | N.A. | N.A. |
| **Page, 2018** | Observational cohort study | 2016-2017 | USA | 170 | MSM | 72.9 | N.A. | N.A. | Mixed | N.A. |
| **Parisi, 2018** | Implementation study | 2014-2015 | USA | 171 | MSM | 93.5 | Daily | N.A. | Mixed | Dependent on insurance |
| **Paulino-Ramirez, 2019** | Observational cohort study | N.A. | DOM | 149 | MSM and TGP | N.A. | N.A. | N.A. | N.A. | N.A. |
| **Pornpaisalsakul, 2020**  (Intervention arm) | Open-label randomized trial | 2019-2020 | THA | 50 | MSM and TGP | 68 | Daily | 18 (17-19) | N.A. | N.A. |
| **Pornpaisalsakul, 2020**  (Standard of care arm) | Open-label randomized trial | 2019-2020 | THA | 50 | MSM and TGP | 64 | Daily | 18 (17-21) | N.A. | N.A. |
| **Reback, 2018** | Observational cohort study | 2016-2018 | USA | 187 | MSM and TGP | 69 | N.A. | N.A. | Mixed | N.A. |
| **Reback, 2019** | Implementation study | 2016-2018 | USA | 187 | MSM and TGP | 69 | Daily | N.A. | Mixed | Dependent on insurance |
| **Refugio, 2019** | Observational cohort study | 2016-2017 | USA | 25 | MSM | 100 | Daily | 22 (18-25) | Mixed | Free of charge |
| **Rusie, 2018** | Observational cohort study | 2012-2017 | USA | 3451 | MSM | 89.1 | N.A. | N.A. | Mixed | Dependent on insurance |
| **Schumacher, 2020** | Observational cohort study | 2015-2018 | USA | 290 | MSM | 100 | N.A. | N.A. | Mixed | N.A. |
| **Selfridge, 2020** | Retrospective cohort study | 2017-2018 | CAN | 124 | MSM | 99.2 | N.A. | N.A. | N.A. | Free of charge |
| **Serota, 2020** | Prospective observational cohort study | 2015-2017 | USA | 131 | MSM | 100 | N.A. | 25 (21-27) | Black | Free of charge |
| **Shover, 2018** | Prospective observational cohort study | 2017-2017 | USA | 1764 | MSM | 94 | N.A. | N.A. | Mixed | N.A. |
| **Songtaweesin, 2020**  (Intervention arm) | Prospective RCT | 2018-2019 | THA | 100 | MSM and TGP | 76 | Daily | 18 (17-19) | N.A. | N.A. |
| **Songtaweesin, 2020**  (Standard of care arm) | Prospective RCT | 2018-2019 | THA | 100 | MSM and TGP | 71 | Daily | 18 (17-19) | N.A. | N.A. |
| **Songtaweesin, 2020** | Observational cohort study | 2018-2019 | THA | 200 | MSM and TGP | 74 | Daily | N.A. | N.A. | Free of charge |
| **Spinelli, 2019** | Observational cohort study | 2012-2017 | USA | 364 | MSM and TGP | 65.9 | N.A. | N.A. | Mixed | Dependent on insurance |
| **Stekler, 2018** | Observational cohort study | 2016-2017 | USA | 48 | MSM | 97.9 | N.A. | N.A. | Mixed | Dependent on insurance |
| **Tan, 2018** | 1-arm, open-label pilot demonstration project | 2014-2014 | CAN | 52 | MSM | 100 | Daily | 33 (28-37) | Mixed | N.A. |
| **Tung, 2017** | Intervention study | 2015-2016 | USA | 245 | MSM | 85.7 | N.A. | N.A. | N.A. | N.A. |
| **Vaccher, 2019** | Demonstration study | 2014-2016 | AUS | 327 | MSM | 100 | Daily | N.A. | Mixed | Free of charge |
| **Veloso, 2019** | Observational cohort study | 2018-2019 | BRA, MEX, PER | 4954 | MSM | 100 | Daily | 29 (24-36) | N.A. | N.A. |
| **Veloso, 2020** | Observational cohort study | 2018-2019 | BRA, MEX, PER | 1843 | MSM | 94 | N.A. | N.A. | Mixed | N.A. |
| **Volk, 2020** | Retrospective cohort study | 2012-2019 | USA | 12,963 | MSM | N.A. | N.A. | N.A. | Mixed | N.A. |
| **Wheeler, 2019** | Demonstration study | 2013-2015 | USA | 178 | MSM | 100 | Daily | 26 (23-32) | Black | N.A. |
| **Wheeler, 2016** | Demonstration study | N.A. | USA | 226 | MSM | 100 | Daily | N.A. | Black | N.A. |
| **Wirtz, 2020** | Observational cohort study | 2015-2020 | THA | 445 | MSM | 93.7 | Daily | N.A. | N.A. | Free of charge |
| **Wu, 2019** | Observational cohort study | 2015-2017 | USA | 333 | MSM and unknown | 73.6 | N.A. | N.A. | Mixed | N.A. |
| **Wu, 2020** | Observational cohort study | 2015-2018 | USA | 412 | MSM | 83.7 | N.A. | 28.7 | Mixed | N.A. |
| **Zablotska, 2018** | Prospective, open-label, single-arm, multicenter demonstration study | 2014-2016 | AUS | 327 | MSM | 97.9 | Daily | 35 (29-43) | Mixed | N.A. |

Abbreviations: MSM=men who have sex with men; TGP=transgender person; PrEP=pre-exposure prophylaxis; IQR=interquartile range; N.A.=not applicable because missing or not reported.
