# Supplementary material for: Oral pre-exposure prophylaxis retention among men who have sex with men and transgender persons: Systematic review and meta-analysis
Source: PLoS One. 2025 Oct 17;20(10):e0333494. doi: 10.1371/journal.pone.0333494 (PMC12533894; doi:10.1371/journal.pone.0333494)
Supplement: S4 Table — Data retrieved from 90 studies conducted between 2010 and 2020; three studies conducted in multiple geographic regions were not included in this analysis. All continuous variables are summarized as median [IQR]. Abbreviations: IQR = interquartile range; MSM = men who have sex with men; TGP = transgender person; PrEP = pre-exposure prophylaxis; STI = sexually transmitted infection. *Defined as the year in which the study with PrEP follow-up was initiated; data missing for 8 studies: North America (n = 4), Australia (n = 1), South America (n = 1), Africa (n = 2). †Defined as the year of last reported follow-up in the study; data missing for 8 studies: North America (n = 4), Australia (n = 1), South America (n = 1), Africa (n = 2). ‡Missing data or nonapplicable study population in 8 studies for MSM [North America (n = 6), South America (n = 2)], 45 for TGP [North America (n = 30), Europe (n = 1), Australia (n = 3), South America (n = 3), Africa (n = 4), Asia (n = 4)] and 63 for other populations [North America (n = 35), Europe (n = 4), Australia (n = 3), South America (n = 6), Africa (n = 5), Asia (n = 10)]. ‖Missing data in 54 studies: North America (n = 37), Australia (n = 2), South America (n = 5), Africa (n = 4), Asia (n = 6). ¶Defined as the terminology used in the study to indicate or determine PrEP retention. $Only one study with reported data. (DOCX) [file pone.0333494.s004.docx]

**S4 Table. Description of study characteristics according to region of study.**

| **Variables** | | **Number of studies**  *n/N (%) or median [IQR]* | | | | | |
| --- | --- | --- | --- | --- | --- | --- | --- |
|  |  | North America  (*N*=53) | Europe  (*N*=5) | Australia  (*N*=6) | South America  (*N*=6) | Africa  (*N*=5) | Asia  (*N*=12) |
| **Calendar year (year)** | |  |  |  |  |  |  |
|  | Year begun * | 2014 [2013-2015] | 2015 [2015-2017] | 2014 [2014-2016] | 2018 [2014-2018] | 2017 [2015-2018] | 2018 [2017-2019] |
|  | Year completed † | 2016 [2015-2017] | 2018 [2017-2019] | 2016 [2016-2017] | 2019 [2016-2019] | 2018 [2016-2019] | 2019 [2019-2020] |
|  | Year published | 2018 [2018-2019] | 2020 [2020-2020] | 2019 [2018-2019] | 2019 [2019-2020] | 2020 [2020-2020] | 2020 [2020-2020] |
| **Percentage of study population (%) ^‡^** | |  |  |  |  |  |  |
|  | MSM | 98.2 [91.0-100] | 99.1 [98.6-99.4] | 99.0 [98.7-100] | 94.4 [94.2-97.2] | 100 [100-100] | 81.0 [72.5-98.0] |
|  | TGP | 4.0 [1.3-8.0] | 0.8 [0.6-1.0] | 0.9 [0.6-1.0] | 5.6 [5.6-6.0] | 21^$^ | 25.0 (10.8-30.50) |
|  | Other | 5.0 [1.8-12.0] | 1.4^$^ | 1.2 [0.1-1.3] | - | - | 8.3 [6.0-10.5] |
| **Geographic setting** | |  |  |  |  |  |  |
|  | Urban | 50 (94.3) | 5 (100) | 5 (83.3) | 6 (100) | 4 (80.0) | 12 (100) |
|  | Urban/rural or rural only | 3 (5.7) | 0 | 1 (16.7) | 0 | 1 (20.0) | 0 |
| **Median study age (year) ^‖^** | | 31 [27-33] | 38 [36-38] | 35 [35-36] | 29^$^ | 24^$^ | 18 [18-21] |
| **Frequency of follow-up visits** | |  |  |  |  |  |  |
|  | <3 months | 44 (86.3) | 2 (50.0) | 4 (66.7) | 6 (100) | 5 (100) | 8 (66.7) |
|  | ≥3 months | 7 (13.7) | 2 (50.0) | 2 (33.3) | 0 | 0 | 4 (33.3) |
| **Study type** | |  |  |  |  |  |  |
|  | Paper | 36 (67.9) | 5 (100) | 4 (66.7) | 2 (33.3) | 2 (40.0) | 6 (50.0) |
|  | Abstract | 17 (32.1) | 0 | 2 (33.3) | 4 (66.7) | 3 (60.0) | 6 (50.0) |
| **PrEP regimen** | |  |  |  |  |  |  |
|  | Daily | 24 (45.3) | 2 (40.0) | 6 (100) | 3 (50.0) | 1 (20.0) | 10 (83.3) |
|  | Event-Driven | 0 | 0 | 0 | 0 | 0 | 1 (33.3) |
|  | Both | 2 (3.8) | 3 (60.0) | 0 | 0 | 0 | 1 (33.3) |
|  | Unspecified | 27 (50.9) | 0 | 0 | 3 (50.0) | 4 (80.0) | 2 (16.7) |
| **PrEP cost** | |  |  |  |  |  |  |
|  | Completely free | 12 (22.6) | 4 (80.0) | 2 (33.3) | 2 (33.3) | 1 (20.0) | 3 (25.0) |
|  | Not entirely free | 7 (13.2) | 0 | 1 (16.7) | 0 | 0 | 2 (16.7) |
|  | Unspecified | 34 (64.2) | 1 (20.0) | 3 (50.0) | 4 (66.7) | 7 (58.3) | 7 (58.3) |
| **STI cost** | |  |  |  |  |  |  |
|  | Unspecified | 48 (90.6) | 5 (100) | 6 (100) | 6 (100) | 4 (80.0) | 11 (91.7) |
|  | Completely free | 0 | 0 | 0 | 0 | 0 | 1 (8.3) |
|  | Not entirely free | 5 (9.4) | 0 | 0 | 0 | 1 (20.0) | 0 |

Data retrieved from 90 studies conducted between 2010 and 2020; three studies conducted in multiple geographic regions were not included in this analysis. All continuous variables are summarized as median [IQR]. Abbreviations: IQR=interquartile range; MSM=men who have sex with men; TGP=transgender person; PrEP=pre-exposure prophylaxis; STI=sexually transmitted infection. *Defined as the year in which the study with PrEP follow-up was initiated; data missing for 8 studies: North America (n=4), Australia (n=1), South America (n=1), Africa (n=2). ^†^Defined as the year of last reported follow-up in the study; data missing for 8 studies: North America (n=4), Australia (n=1), South America (n=1), Africa (n=2). ^‡^Missing data or nonapplicable study population in 8 studies for MSM [North America (n=6), South America (n=2)], 45 for TGP [North America (n=30), Europe (n=1), Australia (n=3), South America (n=3), Africa (n=4), Asia (n=4)] and 63 for other populations [North America (n=35), Europe (n=4), Australia (n=3), South America (n=6), Africa (n=5), Asia (n=10)]. ^‖^Missing data in 54 studies: North America (n=37), Australia (n=2), South America (n=5), Africa (n=4), Asia (n=6). ^¶^Defined as the terminology used in the study to indicate or determine PrEP retention. ^$^Only one study with reported data.
