# Supplementary material for: Oral pre-exposure prophylaxis retention among men who have sex with men and transgender persons: Systematic review and meta-analysis
Source: PLoS One. 2025 Oct 17;20(10):e0333494. doi: 10.1371/journal.pone.0333494 (PMC12533894; doi:10.1371/journal.pone.0333494)
Supplement: S5 Table — Abbreviations: N.A. = not applicable in case of missing/non-reported data. (DOCX) [file pone.0333494.s005.docx]

**S5 Table. Table of primary retention outcomes.**

| **Study**  *(Author, year)* | **Follow-up  interval** *(months)* | **Retention ascertainment** | **Retention terminology** |  | |  | | **Percentage (%) of participants retained in care per month number** | | | | | | | | | | | | | |
| --- | --- | --- | --- | --- | --- | --- | --- | --- | --- | --- | --- | --- | --- | --- | --- | --- | --- | --- | --- | --- | --- |
|  |  |  |  | **3** | **6** | | **9** | | **12** | **15** | **18** | **21** | **24** | **27** | **30** | **33** | **36** | **42** | **48** | **60** |  |
| **Ahaus, 2020** | 2 | Attendance | Follow-up | 82 |  | |  | |  |  |  |  |  |  |  |  |  |  |  |  |  |
| **Akbar, 2020** | 3 | Attendance | Follow-up |  |  | |  | | 92.6 |  |  |  |  |  |  |  |  |  |  |  |  |
| **Akolo, 2020**  (Intervention arm) | 3 | Attendance | Retention | 91.7 | 69 | |  | |  |  |  |  |  |  |  |  |  |  |  |  |  |
| **Akolo, 2020**  (Standard of care arm) | 3 | Attendance | Retention | 31 | 19 | |  | |  |  |  |  |  |  |  |  |  |  |  |  |  |
| **Bhatia, 2021** | 4 | Attendance | Retention | 76.3 | 68.4 | | 65.5 | | 41.7 |  |  |  |  |  |  |  |  |  |  |  |  |
| **Blaylock, 2018** | 1 | Prescription | Discontinuation | 61.6 | 42.1 | |  | |  |  |  |  |  |  |  |  |  |  |  |  |  |
| **Blumenthal, 2017** | 3 | Attendance | Follow-up |  |  | |  | | 78.6 |  |  |  |  |  |  |  |  |  |  |  |  |
| **Chan, 2016** | 3 | Attendance | Retention | 89.2 | 73.4 | |  | |  |  |  |  |  |  |  |  |  |  |  |  |  |
| **Chan, 2019** | 3 | Attendance | Retention | 53.5 |  | |  | | 30 |  |  |  |  |  |  |  |  |  |  |  |  |
| **Chinbunchorn, 2020** | 3 | Attendance | Retention | 49.7 | 42.6 | | 39.1 | | 35 |  |  |  |  |  |  |  |  |  |  |  |  |
| **Clement, 2019** | 3 | Attendance | Persistence | 88.1 |  | |  | |  |  |  |  |  |  |  |  |  |  |  |  |  |
| **Colson, 2020**  (Intervention arm) | 3 | Attendance | Adherence | 66.3 | 66.3 | | 62.4 | | 68.3 |  |  |  |  |  |  |  |  |  |  |  |  |
| **Colson, 2020**  (Standard of care arm) | 3 | Attendance | Follow-up | 62.1 | 59.2 | | 60.2 | | 61.2 |  |  |  |  |  |  |  |  |  |  |  |  |
| **Coyer, 2020** | 3 | Attendance | Discontinuation | 98.4 | 98.1 | | 97.3 | | 95.3 | 93.7 | 91.7 | 90.7 | 89 | 88.2 | 87.1 | 84.6 | 79.6 |  |  |  |  |
| **Doblecki-Lewis, 2018** | 3 | Attendance | Retention | 89.0 |  | |  | |  |  |  |  |  |  |  |  |  |  |  |  |  |
| **Dourado, 2021** | 3 | Attendance | Follow-up | 69.7 |  | |  | |  |  |  |  |  |  |  |  |  |  |  |  |  |
| **Edelman, 2017** | 3 | Attendance | Retention | 71 |  | |  | |  |  |  |  |  |  |  |  |  |  |  |  |  |
| **Egan, 2020** | 3 | Attendance | Follow-up | 77.8 |  | |  | |  |  |  |  |  |  |  |  |  |  |  |  |  |
| **Georgescu, 2017** | 3 | Attendance | Retention | 75 |  | |  | |  |  |  |  |  |  |  |  |  |  |  |  |  |
| **Golub, 2018** | 3 | Attendance | Retention |  |  | |  | | 87 |  |  |  |  |  |  |  |  |  |  |  |  |
| **Grant, 2014** | 3 | Attendance | Adherence | 91.4 | 86.5 | | 82.4 | | 81.4 | 79 |  |  |  |  |  |  |  |  |  |  |  |
| **Grant, 2018** | 3 | Attendance | Retention/follow-up | 94.1 | 89.9 | |  | |  |  |  |  |  |  |  |  |  |  |  |  |  |
| **Greenwald, 2018** | N.A. | Attendance | Retention |  |  | |  | | 52 |  |  |  |  |  |  |  |  |  |  |  |  |
| **Grinsztejn, 2018** | 3 | Attendance | Retention |  |  | |  | | 83.3 |  |  |  |  |  |  |  |  |  |  |  |  |
| **Grulich, 2018** | 3 | Attendance | Follow-up | 90 | 85 | | 79 | | 76 |  |  |  |  |  |  |  |  |  |  |  |  |
| **Havens, 2019** | 3 | N.A. | Retention | 73.3 | 60 | | 43.3 | | 28.3 |  |  |  |  |  |  |  |  |  |  |  |  |
| **Hickey, 2020** | 12 | N.A. | Persistence/retention |  |  | |  | | 75.9 |  |  |  |  |  |  |  |  |  |  |  |  |
| **Hoenigl, 2018** | 3 | Attendance | Completion/follow-up |  |  | |  | | 81.7 |  |  |  |  |  |  |  |  |  |  |  |  |
| **Hojilla, 2018** | 3 | Attendance | Retention/follow-up | 96 | 79 | | 71 | | 62 |  |  |  |  |  |  |  |  |  |  |  |  |
| **Hosek, 2017** | 3 | Attendance | Discontinuation |  |  | |  | | 63.9 |  |  |  |  |  |  |  |  |  |  |  |  |
| **Hoth, 2019** | 3 | Attendance | Retention/follow-up | 86 | 61 | |  | |  |  |  |  |  |  |  |  |  |  |  |  |  |
| **Huang, 2019** | 6 | Prescription | (non) persistence |  | 74.8 | |  | | 55.7 |  |  |  |  |  |  |  |  |  |  |  |  |
| **Hucks-Ortiz, 2016** | 12 | Attendance | Follow-up |  |  | |  | | 92.5 |  |  |  |  |  |  |  |  |  |  |  |  |
| **Iniesta, 2021** | 12 | Attendance | Retention |  |  | |  | | 87.2 |  |  |  |  |  |  |  |  |  |  |  |  |
| **Kaewpoowat, 2019** | 3 | Attendance | Retention |  |  | |  | | 70 |  |  |  |  |  |  |  |  |  |  |  |  |
| **Kimani, 2021** | 3 | Attendance | Follow-up |  | 79.2 | |  | |  |  |  |  |  |  |  |  |  |  |  |  |  |
| **Kyongo, 2018** | 1 | Attendance | Retention | 21.7 | 14.8 | |  | |  |  |  |  |  |  |  |  |  |  |  |  |  |
| **Lal, 2017** | 3 | Attendance | Follow-up | 97.3 | 92.1 | | 93.9 | | 92.1 |  |  |  |  |  |  |  |  |  |  |  |  |
| **Lalley-Chareczko, 2017** | 3 | Attendance | Retention | 90 | 74 | | 70 | | 70 |  |  |  |  |  |  |  |  |  |  |  |  |
| **Landovitz, 2017** | 3 | Attendance | Retention/follow-up | 91.2 | 86.5 | | 82.2 | | 75.1 |  |  |  |  |  |  |  |  |  |  |  |  |
| **Lee, 2019** | 3 | Attendance | Retention |  | 80.3 | |  | |  |  |  |  |  |  |  |  |  |  |  |  |  |
| **Liu, 2019**  (Intervention arm) | 3 | Attendance | Retention | 86 | 81 | | 80 | |  |  |  |  |  |  |  |  |  |  |  |  |  |
| **Liu, 2019**  (Standard arm) | 3 | Attendance | Retention | 75 | 65 | | 57 | |  |  |  |  |  |  |  |  |  |  |  |  |  |
| **Liu, 2015** | 3 | Attendance | Retention |  |  | |  | | 83 |  |  |  |  |  |  |  |  |  |  |  |  |
| **Liu, 2016** | 3 | Attendance | Retention |  |  | |  | | 78 |  |  |  |  |  |  |  |  |  |  |  |  |
| **Marins, 2019** | 3 | Attendance | Retention |  |  | |  | | 83 |  |  |  |  |  |  |  |  |  |  |  |  |
| **McAllister, 2019** | 12 | Attendance | Retention |  |  | |  | |  | 63.8 |  |  |  | 43 |  |  |  |  |  |  |  |
| **Medland, 2020** | 3 | Attendance | Discontinuation |  |  | |  | |  |  | 74.1 |  |  |  |  |  |  |  |  |  |  |
| **Mehrotra, 2021** | 3 | Attendance | Maintenance |  |  | |  | | 64.9 |  |  |  |  |  |  |  |  |  |  |  |  |
| **Mehta, 2020** | 3 | Attendance | Attendance/follow-up |  | 99 | |  | | 94 |  |  |  |  |  |  |  |  |  |  |  |  |
| **Milam, 2019** | 3 | Attendance | Retention |  | 87 | |  | | 81 |  |  |  |  |  |  |  |  |  |  |  |  |
| **Miltz, 2019** | N.A. | Attendance | Attendance/follow-up |  |  | |  | | 76 |  |  |  | 62 |  |  |  |  |  |  |  |  |
| **Molina, 2017** | 2 | Attendance | Attendance/follow-up | 96.1 | 96 | | 93.7 | | 95.7 | 93.7 | 92.5 |  |  |  |  |  |  |  |  |  |  |
| **Montano, 2018** | 3 | Attendance | Discontinuation | 73.2 | 60.1 | | 47.5 | | 34.4 |  |  |  |  |  |  |  |  |  |  |  |  |
| **Montgomery, 2016** | 3 | Attendance | Retention | 62 | 38 | |  | |  |  |  |  |  |  |  |  |  |  |  |  |  |
| **Moore, 2018**  (Intervention arm) | 3 | Attendance | Discontinuation |  |  | |  | | 79 |  |  |  |  |  |  |  |  |  |  |  |  |
| **Moore, 2018**  (Standard of care arm) | 3 | Attendance | Discontinuation |  |  | |  | | 83.8 |  |  |  |  |  |  |  |  |  |  |  |  |
| **Newcomb, 2019** | 6 | Attendance | Discontinuation |  |  | |  | | 87.4 |  |  |  |  |  |  |  |  | 80 |  |  |  |
| **Nguyen, 2018** | 3 | Attendance | Discontinuation |  |  | |  | | 75.2 |  |  |  |  |  |  |  |  |  |  |  |  |
| **Nostlinger, 2020** | 9 | Attendance | Retention |  |  | | 93 | |  |  | 89.5 |  |  |  |  |  |  |  |  |  |  |
| **Page, 2018** | 6 | Attendance | Retention |  | 52.9 | |  | | 28.9 |  |  |  |  |  |  |  |  |  |  |  |  |
| **Parisi, 2018** | 3 | Attendance | Discontinuation |  |  | |  | |  |  | 76 |  |  |  |  |  |  |  |  |  |  |
| **Paulino-Ramirez, 2019** | 3 | Attendance | Persistence in care | 93.1 |  | |  | |  |  |  |  |  |  |  |  |  |  |  |  |  |
| **Pornpaisalsakul, 2020**  (Intervention arm) | 6 | Attendance | Follow-up |  | 74 | |  | |  |  |  |  |  |  |  |  |  |  |  |  |  |
| **Pornpaisalsakul, 2020**  (Standard of care arm) | 6 | Attendance | Follow-up |  | 84 | |  | |  |  |  |  |  |  |  |  |  |  |  |  |  |
| **Reback, 2018** | 3 | Attendance | Follow-up | 74.6 |  | |  | |  |  |  |  |  |  |  |  |  |  |  |  |  |
| **Reback, 2019** | 3 | Attendance | Follow-up | 88.2 |  | |  | |  |  |  |  |  |  |  |  |  |  |  |  |  |
| **Refugio, 2019** | 3 | Attendance | Retention |  | 84 | |  | |  |  |  |  |  |  |  |  |  |  |  |  |  |
| **Rusie, 2018** | 3 | Attendance | Retention | 89.2 | 89.6 | | 80 | | 56.8 |  |  |  |  |  |  |  |  |  |  |  |  |
| **Schumacher, 2020** | 6 | Attendance | Follow-up/return for care |  | 73.4 | |  | | 44.8 |  |  |  |  |  |  |  |  |  |  |  |  |
| **Selfridge, 2020** | 3 | Prescription | Follow-up | 85.5 | 79.8 | |  | |  |  |  |  |  |  |  |  |  |  |  |  |  |
| **Serota, 2020** | 3 | Attendance | Discontinuation | 91.6 | 79.4 | | 70.2 | | 62.6 | 51.1 | 38.9 | 26.7 | 0.8 |  |  |  |  |  |  |  |  |
| **Shover, 2018** | 3 | Attendance | Discontinuation | 32 | 45 | |  | |  |  |  |  |  |  |  |  |  |  |  |  |  |
| **Songtaweesin, 2020**  (Intervention arm) | 1 | Attendance | Retention |  | 73 | |  | |  |  |  |  |  |  |  |  |  |  |  |  |  |
| **Songtaweesin, 2020**  (Standard of care arm) | 1 | Attendance | Retention |  | 72 | |  | |  |  |  |  |  |  |  |  |  |  |  |  |  |
| **Songtaweesin, 2020** | 1 | Attendance | Retention | 86 | 75 | |  | |  |  |  |  |  |  |  |  |  |  |  |  |  |
| **Spinelli, 2019** | 3 | Attendance | Discontinuation | 84.1 |  | |  | | 37.9 |  |  |  |  |  |  |  |  |  |  |  |  |
| **Stekler, 2018** | 3 | Attendance | Follow-up | 59.5 |  | |  | |  |  |  |  |  |  |  |  |  |  |  |  |  |
| **Tan, 2018** | 3 | Attendance | Retention | 98.1 | 96.2 | | 88.5 | | 82.7 |  |  |  |  |  |  |  |  |  |  |  |  |
| **Tung, 2017** | N.A. | Prescription | Retention |  |  | |  | | 75 |  |  |  |  |  |  |  |  |  |  |  |  |
| **Vaccher, 2019** | 3 | Attendance | Retention | 95.4 | 92 | | 88.4 | | 81 |  |  |  |  |  |  |  |  |  |  |  |  |
| **Veloso, 2019** | 3 | Attendance | Early continuation | 79.8 |  | |  | |  |  |  |  |  |  |  |  |  |  |  |  |  |
| **Veloso, 2020** | 3 | Attendance | Early continuation |  | 67.2 | |  | |  |  |  |  |  |  |  |  |  |  |  |  |  |
| **Volk, 2020** | 12 | Prescription | Persistence |  |  | |  | | 73 |  |  |  | 64 |  |  |  | 60 |  | 57 | 56 |  |
| **Wheeler, 2019** | 3 | Attendance | Retention | 96.1 | 94,9 | | 93.8 | | 96.1 |  |  |  |  |  |  |  |  |  |  |  |  |
| **Wheeler, 2016** | 12 | Attendance | Follow-up |  |  | |  | | 92.5 |  |  |  |  |  |  |  |  |  |  |  |  |
| **Wirtz, 2020** | 3 | Attendance | Retention |  |  | |  | | 94.8 |  |  |  |  |  |  |  |  |  |  |  |  |
| **Wu, 2019** | 3 | Attendance | Discontinuation | 77.2 |  | |  | | 49.5 |  |  |  |  |  |  |  |  |  |  |  |  |
| **Wu, 2020** | 3 | Attendance | Retention | 76.4 | 57.5 | | 49.6 | | 47.2 |  |  |  |  |  |  |  |  |  |  |  |  |
| **Zablotska, 2018** | 6 | Attendance | Retention | 95.7 | 92 | | 88.7 | | 81 | 68.5 | 34.3 | 5.5 |  |  |  |  |  |  |  |  |  |

Abbreviations: N.A.=not applicable in case of missing/non-reported data.
