# Supplementary figures and images for: Oral pre-exposure prophylaxis retention among men who have sex with men and transgender persons: Systematic review and meta-analysis
Source: PLoS One. 2025 Oct 17;20(10):e0333494. doi: 10.1371/journal.pone.0333494 (PMC12533894; doi:10.1371/journal.pone.0333494)

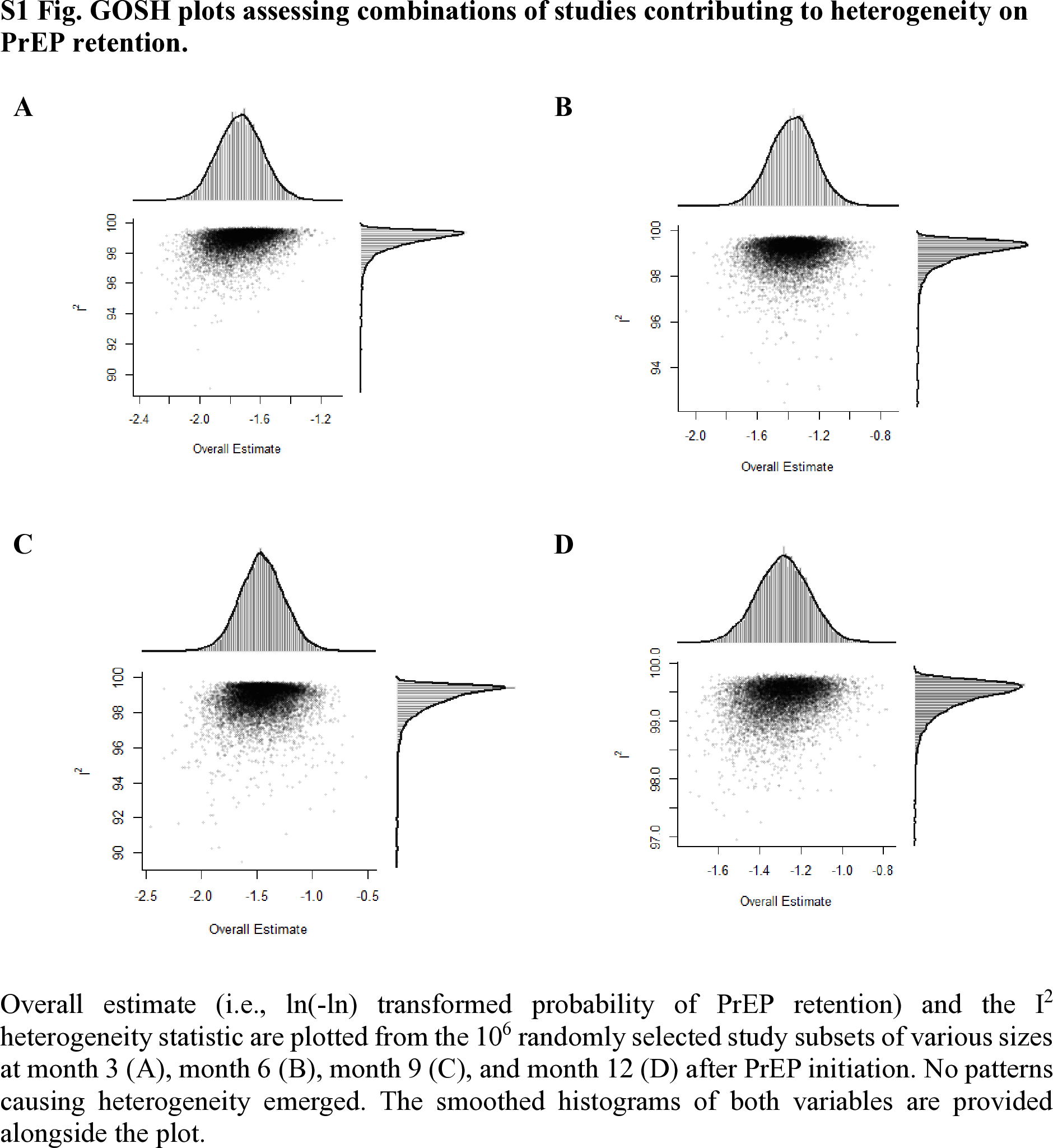

Supplement: S1 Fig — Observed outcome (i.e., ln(-ln) transformed probability of PrEP retention) and the I2 heterogeneity statistic are plotted from the 10,000 randomly selected study subsets of various sizes at month 3 (A), month 6 (B), month 9 (C), and month 12 (D) after PrEP initiation. No patterns causing heterogeneity emerged. The smoothed histograms of both variables are provided alongside the plot. (TIF) [file pone.0333494.s006.tif]

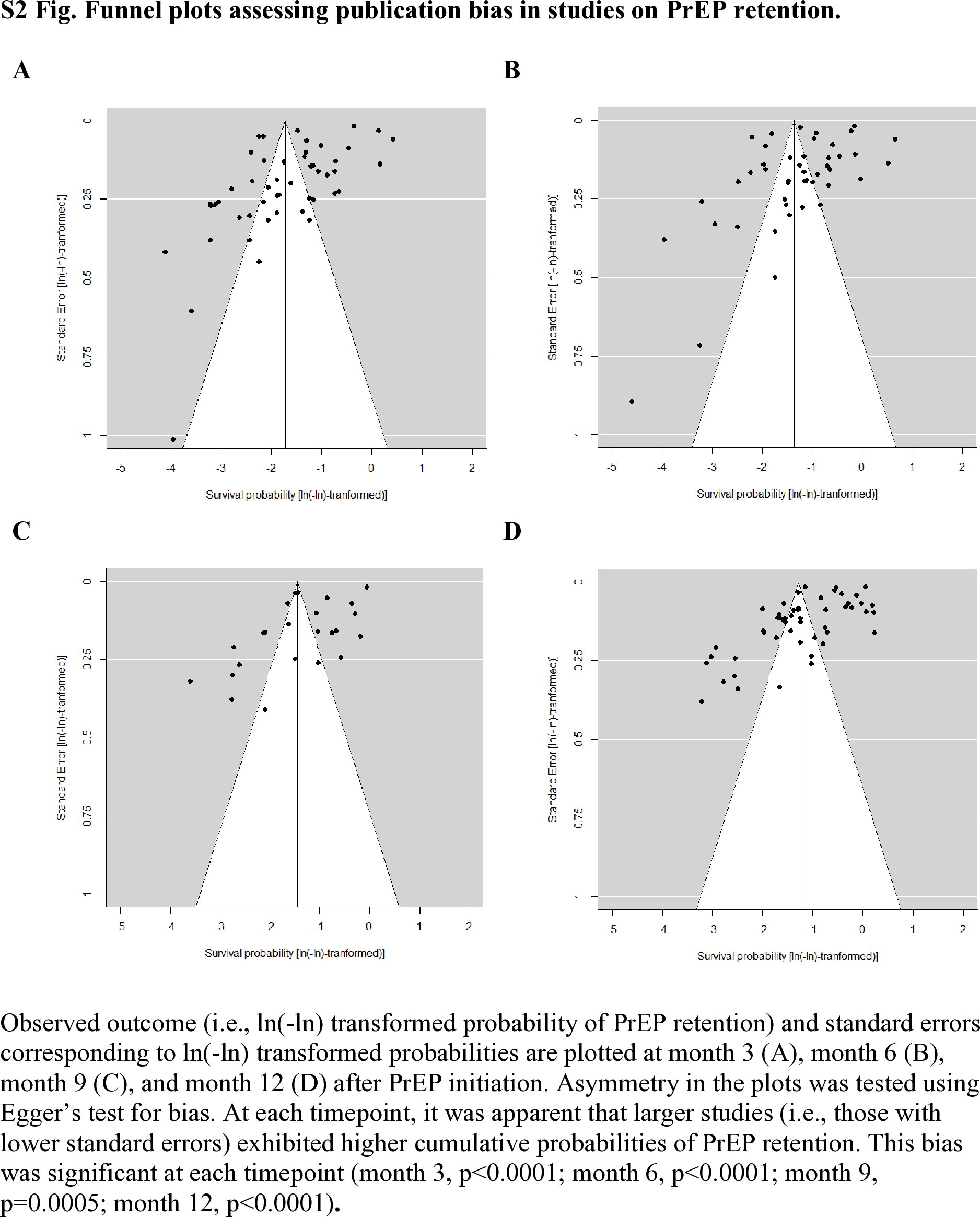

Supplement: S2 Fig — Observed outcome (i.e., ln(-ln) transformed probability of PrEP retention) and standard errors corresponding to ln(-ln) transformed probabilities are plotted at month 3 (A), month 6 (B), month 9 (C), and month 12 (D) after PrEP initiation. Asymmetry in the plots was tested using Egger’s test for bias. At each timepoint, it was apparent that larger studies (i.e., those with lower standard errors) exhibited higher cumulative probabilities of PrEP retention. This bias was significant at each timepoint (month 3, p < 0.0001; month 6, p < 0.0001; month 9, p = 0.0005; month 12, p < 0.0001). (TIF) [file pone.0333494.s007.tif]
