# Supplementary material for: Oral pre-exposure prophylaxis retention among men who have sex with men and transgender persons: Systematic review and meta-analysis
Source: PLoS One. 2025 Oct 17;20(10):e0333494. doi: 10.1371/journal.pone.0333494 (PMC12533894; doi:10.1371/journal.pone.0333494)
Supplement: S1 References — References of included papers and abstracts (cited in S2 Table). (DOCX) [file pone.0333494.s008.docx]

**S1 References. References of included papers and abstracts (cited in S2 Table).**

1. Ahaus P, Potthoff A, Kayser A, Wach J, Brockmeyer NH, Skaletz-Rorowski A. HIV pre-exposure prophylaxis care in intersectoral collaboration: Interim analysis of a monocentric, prospective study in Germany. Hautarzt. 2020;71(3):211-8.

2. Akbar M, Azwa I, Chong M, Hangchen O, Basri S, Yap A, et al. Project My PrEP: Results from a PrEP demonstration project among high-risk men who have sex with men (MSM) in Kuala Lumpur, Malaysia. Journal of the International AIDS Society. 2020;23(SUPPL 4).

3. Akolo M, Kimani J, Gichuki R, Osero J, Kibera A, Gelmon L. Effect of targeted counselling on retention to HIV pre- exposure prophylaxis among men who have sex with men within Nairobi City County, Kenya. Journal of the International AIDS Society. 2020;23(SUPPL 4).

4. Bhatia R, Khoa TCD, Hao BTM, Van HTH, Hieu TT, Ngoc LB, et al. Initial Results of PrEP Implementation in a MSM Sexual Health Clinic in Hanoi, Vietnam. Journal of the International AIDS Society. 2021;24(SUPPL 1):135-6.

5. Blaylock JM, Hakre S, Decker CF, Wilson B, Bianchi E, Michael N, et al. HIV PrEP in the Military: Experience at a Tertiary Care Military Medical Center. Military medicine. 2018;183(1 Supplement):445-9.

6. Blumenthal J, Jain S, Dube M, Sun X, Ellorin E, Hoenigl M, et al. Recent HIV risk behavior and partnership type predict prep adherence in men who have sex with men. Open Forum Infectious Diseases. 2017;4(Supplement 1):S15-S6.

7. Chan PA, Glynn TR, Oldenburg CE, Montgomery MC, Robinette AE, Almonte A, et al. Implementation of Preexposure Prophylaxis for Human Immunodeficiency Virus Prevention Among Men Who Have Sex With Men at a New England Sexually Transmitted Diseases Clinic. Sex Transm Dis. 2016;43(11):717-23.

8. Chan PA, Patel RR, Mena L, Marshall BDL, Rose J, Sutten Coats C, et al. Long-term retention in pre-exposure prophylaxis care among men who have sex with men and transgender women in the United States. Journal of the International AIDS Society. 2019;22(8):e25385.

9. Chinbunchorn T, Nampaisarn O, Lujintanon S, Janyam S, Sangprasert T, Chanlearn P, et al. Factors associated with loss to retention among free and feebased prep in thailand. Topics in Antiviral Medicine. 2020;28(1):381.

10. Clement ME, Johnston BE, Eagle C, Taylor D, Rosengren AL, Goldstein BA, et al. Advancing the HIV Pre-Exposure Prophylaxis Continuum: A Collaboration between a Public Health Department and a Federally Qualified Health Center in the Southern United States. AIDS Patient Care and STDs. 2019;33(8):366-71.

11. Colson PW, Franks J, Wu Y, Winterhalter FS, Knox J, Ortega H, et al. Adherence to Pre-exposure Prophylaxis in Black Men Who Have Sex with Men and Transgender Women in a Community Setting in Harlem, NY. AIDS and behavior. 2020;24(12):3436-55.

12. Coyer L, van den Elshout MAM, Achterbergh RCA, Matser A, Schim van der Loeff MF, Davidovich U, et al. Understanding pre-exposure prophylaxis (PrEP) regimen use: Switching and discontinuing daily and event-driven PrEP among men who have sex with men. EClinicalMedicine. 2020;29-30:100650.

13. Doblecki-Lewis S, Liu AY, Feaster DJ, Cohen SE, Elion R, Bacon O, et al. Patterns and Correlates of Participant Retention in a Multi-City Pre-Exposure Prophylaxis Demonstration Project. Journal of acquired immune deficiency syndromes (1999). 2018;79(1):62-9.

14. Dourado I, Magno L, Soares F, Caires P, Eustorgio M, Tupinambas U, et al. PrEP initiation and continuation among adolescent key population in Brazil: A cascade analysis. Journal of the International AIDS Society. 2021;24(SUPPL 1):142-3.

15. Edelman EJ, Ogbuagu O, Williams E, Barakat LA, Ogunbajo A, Tiberio PJ, et al. The prevalence and impact of unhealthy alcohol use among men who have sex with men initiating pre-exposure prophylaxis for HIV infection: A pilot study. Journal of General Internal Medicine. 2017;32(2 Supplement 1):S348-S9.

16. Egan JE, Ho K, Stall R, Drucker MT, Tappin R, Hendrix CW, et al. Feasibility of Short-Term PrEP Uptake for Men Who Have Sex with Men with Episodic Periods of Increased HIV Risk. Journal of Acquired Immune Deficiency Syndromes. 2020;84(5):508-13.

17. Georgescu A, Egurrola C, Schaff S, Fisher J, Smith S, Florita C, et al. Prep uptake and emergent HIV infections in Southern Arizona: Is there a disconnect? Open Forum Infectious Diseases. 2017;4(Supplement 1):S438-S9.

18. Golub SA, Enemchukwu CU. The critical importance of retention in HIV prevention. Lancet HIV. 2018;5(9):e475-e6.

19. Grant RM, Anderson PL, McMahan V, Liu A, Amico KR, Mehrotra M, et al. Uptake of pre-exposure prophylaxis, sexual practices, and HIV incidence in men and transgender women who have sex with men: A cohort study. The Lancet Infectious Diseases. 2014;14(9):820-9.

20. Grant RM, Mannheimer S, Hughes JP, Hirsch-Moverman Y, Loquere A, Chitwarakorn A, et al. Daily and Nondaily Oral Preexposure Prophylaxis in Men and Transgender Women Who Have Sex With Men: The Human Immunodeficiency Virus Prevention Trials Network 067/ADAPT Study. Clinical infectious diseases : an official publication of the Infectious Diseases Society of America. 2018;66(11):1712-21.

21. Greenwald Z, Card K, Niaki N, Lachowsky N, Thomas R. Geographic barriers result in HIV pre-exposure prophylaxis discontinuation: How to improve retention in care. Journal of the International AIDS Society. 2018;21(Supplement 8):26.

22. Grinsztejn B, Hoagland B, Moreira RI, Kallas EG, Madruga JV, Goulart S, et al. Retention, engagement, and adherence to pre-exposure prophylaxis for men who have sex with men and transgender women in PrEP Brasil: 48 week results of a demonstration study. The lancet HIV. 2018;5(3):e136-e45.

23. Grulich AE, Guy R, Amin J, Jin F, Selvey C, Holden J, et al. Population-level effectiveness of rapid, targeted, high-coverage roll-out of HIV pre-exposure prophylaxis in men who have sex with men: the EPIC-NSW prospective cohort study. The lancet HIV. 2018;5(11):e629-e37.

24. Havens JP, Scarsi KK, Sayles H, Klepser DG, Swindells S, Bares SH. Acceptability and Feasibility of a Pharmacist-Led Human Immunodeficiency Virus Pre-Exposure Prophylaxis Program in the Midwestern United States. Open Forum Infectious Diseases. 2019;6(10):ofz365.

25. Hickey A, Weir B, Dun C, Wirtz A, Mon SHH, Chemnasiri T, et al. High persistence of daily oral PrEP among 18 to 26 year old Thai men who sell sex: Preliminary results of the COPE4YMSM study. Journal of the International AIDS Society. 2020;23(SUPPL 4).

26. Hoenigl M, Jain S, Moore D, Collins D, Sun X, Anderson PL, et al. Substance use and adherence to HIV preexposure prophylaxis for men who have sex with men. Emerging Infectious Diseases. 2018;24(12):2292-302.

27. Hojilla JC, Vlahov D, Crouch PC, Dawson-Rose C, Freeborn K, Carrico A. HIV Pre-exposure Prophylaxis (PrEP) Uptake and Retention Among Men Who Have Sex with Men in a Community-Based Sexual Health Clinic. AIDS and behavior. 2018;22(4):1096-9.

28. Hosek SG, Landovitz RJ, Kapogiannis B, Siberry GK, Rudy B, Rutledge B, et al. Safety and feasibility of antiretroviral preexposure prophylaxis for adolescent men who have sex with men aged 15 to 17 years in the United States. JAMA Pediatrics. 2017;171(11):1063-71.

29. Hoth AB, Shafer C, Dillon DB, Mayer R, Walton G, Ohl ME. Iowa TelePrEP: A Public-Health-Partnered Telehealth Model for Human Immunodeficiency Virus Preexposure Prophylaxis Delivery in a Rural State. Sexually transmitted diseases. 2019;46(8):507-12.

30. Huang W, Wu D, Ong JJ, Smith MK, Pan S, Yang F, et al. Prepared for PrEP: preferences for HIV pre-exposure prophylaxis among Chinese men who have sex with men in an online national survey. BMC Infect Dis. 2019;19(1):1057.

31. Hucks-Ortiz C, Lucas JP, Wheeler DP, Fields SD. HPTN 073: Successful engagement of Black MSM into a culturally relevant clinical trial for pre-exposure prophylaxis. Journal of the International AIDS Society. 2016;19(Supplement 5):42-3.

32. Iniesta C, Coll P, Barbera MJ, Deltoro MG, Camino X, Fagundez G, et al. Implementation of pre-exposure prophylaxis programme in Spain. Feasibility of four different delivery models. PLoS ONE. 2021;16(2 February):e0246129.

33. Kaewpoowat Q, Saokhieo P, Songsupa R, Supindham T, Chariyalertsak S. Prep acceptability, uptake, and adherence among young men who have sex with men and transgender women in PrEP demonstration project, Chiang Mai, Thailand. Open Forum Infectious Diseases. 2019;6(Supplement 2):S462-S3.

34. Kimani M, van der Elst EM, Chirro O, Wahome E, Ibrahim F, Mukuria N, et al. "I wish to remain HIV negative": Pre-exposure prophylaxis adherence and persistence in transgender women and men who have sex with men in coastal Kenya. PLoS One. 2021;16(1):e0244226.

35. Kyongo JK, Kiragu M, Karuga R, Ochieng C, Ngunjiri A, Wachihi C, et al. How long will they take it? Oral pre-exposure prophylaxis (PrEP) retention for female sex workers, men who have sex with men and young women in a demonstration project in Kenya. Journal of the International AIDS Society. 2018;21(Supplement 6).

36. Lal L, Audsley J, Murphy DA, Fairley CK, Stoove M, Roth N, et al. Medication adherence, condom use and sexually transmitted infections in Australian preexposure prophylaxis users. AIDS. 2017;31(12):1709-14.

37. Lalley-Chareczko L, Clark D, Moorthy GS, Zuppa A, Conyngham C, Mounzer K, et al. Urine tenofovir testing to measure PrEP adherence among youth in a real world setting. Topics in Antiviral Medicine. 2017;25(1 Supplement 1):412s-3s.

38. Landovitz RJ, Beymer M, Kofron R, Amico KR, Psaros C, Bushman L, et al. Plasma tenofovir levels to support adherence to TDF/FTC preexposure prophylaxis for HIV prevention in MSM in Los Angeles, California. Journal of Acquired Immune Deficiency Syndromes. 2017;76(5):501-11.

39. Lee SS, Kwan TH, Wong NS, Lee KCK, Chan DPC, Lam TTN, et al. Piloting a partially self-financed mode of human immunodeficiency virus pre-exposure prophylaxis delivery for men who have sex with men in hong kong. Hong Kong Medical Journal. 2019;25(5):382-91.

40. Liu A, Cohen S, Vittinghoff E, Anderson P, Doblecki-Lewis S, Bacon O, et al. Adherence, sexual behaviour and HIV/STI incidence among men who have sex with men and transgender women in the US PrEP demonstration (Demo) project. Journal of the International AIDS Society. 2015;18(SUPPL. 4):46-7.

41. Liu AY, Cohen SE, Vittinghoff E, Anderson PL, Doblecki-Lewis S, Bacon O, et al. Preexposure prophylaxis for HIV infection integrated with municipal-and community-based sexual health services. JAMA Internal Medicine. 2016;176(1):75-84.

42. Liu AY, Vittinghoff E, von Felten P, Rivet Amico K, Anderson PL, Lester R, et al. Randomized Controlled Trial of a Mobile Health Intervention to Promote Retention and Adherence to Preexposure Prophylaxis Among Young People at Risk for Human Immunodeficiency Virus: The EPIC Study. Clinical infectious diseases : an official publication of the Infectious Diseases Society of America. 2019;68(12):2010-7.

43. Marins LMS, Torres TS, Leite IdC, Moreira RI, Luz PM, Hoagland B, et al. Performance of HIV pre-exposure prophylaxis indirect adherence measures among men who have sex with men and transgender women: Results from the PrEP Brasil Study. PloS one. 2019;14(8):e0221281.

44. McAllister J, Holliday S, Richardson R, Hesse K, Comben S, Carr A. Changes in bone mineral density over 2 years in men who have sex with men on tenofovir disoproxil fumarate-based HIV pre-exposure prophylaxis: Longitudinal cohort data. Antiviral Therapy. 2019;24(Supplement 1):A21.

45. Medland N, Guy R, Grulich A, Bavinton B, Keen P, Ellard J, et al. Successful national PrEP scale-up in Australia: Evaluation of uptake, adherence, discontinuation and HIV seroconversion from April 2018 to September 2019 using national dispensing data. Journal of the International AIDS Society. 2020;23(SUPPL 4).

46. Mehrotra ML, Westmoreland DA, Patel VV, Hojilla JC, Grov C. Breaking Inertia: Movement Along the PrEP Cascade in a Longitudinal US National Cohort of Sexual Minority Individuals at Risk for HIV. Journal of acquired immune deficiency syndromes (1999). 2021;86(5):e118-e25.

47. Mehta SD, Okall D, Graham SM, N'Gety G, Bailey RC, Otieno F. Behavior Change and Sexually Transmitted Incidence in Relation to PREP Use Among Men Who Have Sex with Men in Kenya. AIDS Behav. 2021.

48. Milam J, Jain S, Dube MP, Daar ES, Sun X, Corado K, et al. Sexual Risk Compensation in a Pre-exposure Prophylaxis Demonstration Study among Individuals at Risk of HIV. Journal of Acquired Immune Deficiency Syndromes. 2019;80(1):E9-E13.

49. Miltz A, Lampe F, McCormack S, Dunn D, White E, Rodger A, et al. Prevalence and correlates of depressive symptoms among gay, bisexual and other men who have sex with men in the PROUD randomised clinical trial of HIV pre-exposure prophylaxis. BMJ Open. 2019;9(12):e031085.

50. Molina JM, Charreau I, Spire B, Cotte L, Chas J, Capitant C, et al. Efficacy, safety, and effect on sexual behaviour of on-demand pre-exposure prophylaxis for HIV in men who have sex with men: an observational cohort study. Lancet HIV. 2017;4(9):e402-e10.

51. Montano MA, Dombrowski JC, Dasgupta S, Golden MR, Duerr A, Manhart LE, et al. Changes in Sexual Behavior and STI Diagnoses Among MSM Initiating PrEP in a Clinic Setting. AIDS and behavior. 2019;23(2):548-55.

52. Montgomery MC, Oldenburg CE, Nunn AS, Mena L, Anderson P, Liegler T, et al. Adherence to pre-Exposure prophylaxis for HIV prevention in a clinical setting. PLoS ONE. 2016;11(6):e0157742.

53. Moore DJ, Jain S, Dube MP, Daar ES, Sun X, Young J, et al. Randomized Controlled Trial of Daily Text Messages to Support Adherence to Preexposure Prophylaxis in Individuals at Risk for Human Immunodeficiency Virus: The TAPIR Study. Clinical Infectious Diseases. 2018;66(10):1566-72.

54. Newcomb ME, Morgan E, Feinstein B, Mustanski B. Longitudinal predictors of prep discontinuation among ymsm and transgender women. Topics in Antiviral Medicine. 2019;27(SUPPL 1):386s.

55. Nguyen VK, Greenwald ZR, Trottier H, Cadieux M, Goyette A, Beauchemin M, et al. Incidence of sexually transmitted infections before and after preexposure prophylaxis for HIV. AIDS. 2018;32(4):523-30.

56. Nostlinger C, Reyniers T, Smekens T, Apers H, Laga M, Wouters K, et al. Drug use, depression and sexual risk behaviour: a syndemic among early pre-exposure prophylaxis (PrEP) adopters in Belgium? AIDS care. 2020;32(sup2):57-64.

57. Page K, Akolo O, Redd R, Kelley E, Dawkins J, Olawale A, et al. Baseline sexually transmitted infections (STI) and patient retention among patients enrolling in PrEP in the baltimore city health department sexual health clinic. Sexually Transmitted Diseases. 2018;45(Supplement 2):S64.

58. Parisi D, Warren B, Leung SJ, Akkaya-Hocagil T, Qin Q, Hahn I, et al. A Multicomponent Approach to Evaluating a Pre-exposure Prophylaxis (PrEP) Implementation Program in Five Agencies in New York. The Journal of the Association of Nurses in AIDS Care : JANAC. 2018;29(1):10-9.

59. Paulino-Ramirez R, Tapia L, Sanchez E, Benitez A, Marino A. HPV-related malignancies screening to strengthen persistence in pre-exposure prophylaxis among MSM and transgender women in the Dominican Republic. Journal of the International AIDS Society. 2019;22(Supplement 2).

60. Pornpaisalsakul K, Songtaweesin WN, Tepmongkol S, Wongharn P, Kawichai S, Suponsilchai V, et al. Effects of vitamin D and calcium supplementation on bone mineral density among Thai youth using daily HIV pre-exposure prophylaxis. Journal of the International AIDS Society. 2020;23(10):e25624.

61. Reback C, Runger D, Fletcher J. Providing PrEP navigation to high-risk populations with multiple health disparities in Los Angeles, CA, USA: A comparison of MSM and trans women. AIDS Research and Human Retroviruses. 2018;34(Supplement 1):390.

62. Reback CJ, Clark KA, Rünger D, Fehrenbacher AE. A Promising PrEP Navigation Intervention for Transgender Women and Men Who Have Sex with Men Experiencing Multiple Syndemic Health Disparities. J Community Health. 2019;44(6):1193-203.

63. Refugio ON, Kimble MM, Silva CL, Lykens JE, Bannister C, Klausner JD. Brief Report: PrEPTECH: A Telehealth-Based Initiation Program for HIV Pre-exposure Prophylaxis in Young Men of Color Who Have Sex with Men. A Pilot Study of Feasibility. Journal of Acquired Immune Deficiency Syndromes. 2019;80(1):40-5.

64. Rusie LK, Orengo C, Burrell D, Ramachandran A, Houlberg M, Keglovitz K, et al. Preexposure Prophylaxis Initiation and Retention in Care Over 5 Years, 2012-2017: Are Quarterly Visits Too Much? Clin Infect Dis. 2018;67(2):283-7.

65. Schumacher C, Wu L, Chandran A, Fields E, Price A, Greenbaum A, et al. Sexually Transmitted Infection Screening Among Gay, Bisexual, and Other Men Who Have Sex With Men Prescribed Pre-exposure Prophylaxis in Baltimore City, Maryland. Clin Infect Dis. 2020;71(10):2637-44.

66. Selfridge M, Card KG, Lundgren K, Barnett T, Guarasci K, Drost A, et al. Exploring nurse-led HIV Pre-Exposure Prophylaxis in a community health care clinic. Public Health Nursing. 2020;37(6):871-9.

67. Serota DP, Rosenberg ES, Sullivan PS, Thorne AL, Rolle CM, Del Rio C, et al. Pre-exposure Prophylaxis Uptake and Discontinuation Among Young Black Men Who Have Sex With Men in Atlanta, Georgia: A Prospective Cohort Study. Clin Infect Dis. 2020;71(3):574-82.

68. Shover CL, Javanbakht M, Shoptaw S, Bolan R, Gorbach P. High discontinuation of pre-exposure prophylaxis within six months of initiation. Topics in Antiviral Medicine. 2018;26(Supplement 1):460s-1s.

69. Songtaweesin WN, Kawichai S, Phanuphak N, Cressey TR, Wongharn P, Saisaengjan C, et al. Youth-friendly services and a mobile phone application to promote adherence to pre-exposure prophylaxis among adolescent men who have sex with men and transgender women at-risk for HIV in Thailand: a randomized control trial. J Int AIDS Soc. 2020;23 Suppl 5(Suppl 5):e25564.

70. Songtaweesin WN, Puthanakit T, Kawichai S, Cressey TR, Wongharn P, Theerawit T, et al. High prep adherence based on TFV-DP levels in THAI 15-19-year-old MSM and transwomen. Topics in Antiviral Medicine. 2020;28(1):387.

71. Spinelli MA, Scott HM, Vittinghoff E, Liu AY, Gonzalez R, Morehead-Gee A, et al. Missed Visits Associated With Future Preexposure Prophylaxis (PrEP) Discontinuation Among PrEP Users in a Municipal Primary Care Health Network. Open Forum Infect Dis. 2019;6(4):ofz101.

72. Stekler JD, McMahan V, Ballinger L, Viquez L, Swanson F, Stockton J, et al. HIV Pre-exposure Prophylaxis Prescribing Through Telehealth. J Acquir Immune Defic Syndr. 2018;77(5):e40-e2.

73. Tan DHS, Schnubb A, Lawless J, Szadkowski L, Grennan T, Wilton J, et al. Acceptability and tolerability of and adherence to HIV preexposure prophylaxis among Toronto gay and bisexual men: a pilot study. CMAJ Open. 2018;6(4):E611-e7.

74. Tung E, Thomas A, Eichner A, Shalit P. Feasibility of a pharmacist-run HIV PrEP clinic in a community pharmacy setting. Topics in Antiviral Medicine. 2017;25(1 Supplement 1):407s.

75. Vaccher SJ, Marzinke MA, Templeton DJ, Haire BG, Ryder N, McNulty A, et al. Predictors of Daily Adherence to HIV Pre-exposure Prophylaxis in Gay/Bisexual Men in the PRELUDE Demonstration Project. AIDS and behavior. 2019;23(5):1287-96.

76. Veloso VG, Vega-Ramirez EH, Hoagland B, Konda KA, Bautista-Arredondo S, Guanira JV, et al. Safety, early continuation and adherence of same day PrEP initiation among MSM and TGW in Brazil, Mexico and Peru: The ImPrEP Study. Journal of the International AIDS Society. 2019;22(Supplement 5).

77. Veloso VG, Vega-Ramirez EH, Hoagland B, Konda KA, Bautista-Arredondo S, Guanira JV, et al. Factors associated with early continuation (EC) of preexposure prophylaxis (PrEP) among young MSM (YMSM) in Brazil, Peru and Mexico: The ImPrEP Study. Journal of the International AIDS Society. 2020;23(SUPPL 4).

78. Volk J, Hojilla JC, Hurley L, Silverberg MJ, Skarbinski J, Satre DD, et al. PrEP continuum of care and new HIV infections: Long‐term follow‐up in a large clinical cohort. J Int AIDS Soc. 2020;23.

79. Wheeler DP, Fields S, Nelson LE, Wilton L, Hightow-Weidman L, Shoptaw S, et al. HPTN 073: Prep uptake and use by black men who have sex with men in 3 us cities. Topics in Antiviral Medicine. 2016;24(E-1):374.

80. Wheeler DP, Fields SD, Beauchamp G, Chen YQ, Emel LM, Hightow-Weidman L, et al. Pre-exposure prophylaxis initiation and adherence among Black men who have sex with men (MSM) in three US cities: results from the HPTN 073 study. J Int AIDS Soc. 2019;22(2):e25223.

81. Wirtz AL, Weir BW, Mon SHH, Sirivongrangson P, Chemnasiri T, Dunne EF, et al. Testing the Effectiveness and Cost-Effectiveness of a Combination HIV Prevention Intervention Among Young Cisgender Men Who Have Sex With Men and Transgender Women Who Sell or Exchange Sex in Thailand: Protocol for the Combination Prevention Effectiveness Study. JMIR Res Protoc. 2020;9(1):e15354.

82. Wu L, Schumacher C, Chandran A, Fields E, Davis M, Ryscavage P, et al. Patterns of HIV pre-exposure prophylaxis care one year after initiating PrEP, Baltimore City, Maryland 2015-2018. Sexually Transmitted Infections. 2019;95(Supplement 1):A61-A2.

83. Wu L, Schumacher C, Chandran A, Fields E, Price A, Greenbaum A, et al. Patterns of PrEP Retention Among HIV Pre-exposure Prophylaxis Users in Baltimore City, Maryland. Journal of acquired immune deficiency syndromes (1999). 2020;85(5):593-600.

84. Zablotska IB, Vaccher SJ, Bloch M, Carr A, Foster R, Grulich AE, et al. High Adherence to HIV Pre-exposure Prophylaxis and No HIV Seroconversions Despite High Levels of Risk Behaviour and STIs: The Australian Demonstration Study PrELUDE. AIDS Behav. 2019;23(7):1780-9.
