# Supplementary material for: Oral pre-exposure prophylaxis retention among men who have sex with men and transgender persons: Systematic review and meta-analysis
Source: PLoS One. 2025 Oct 17;20(10):e0333494. doi: 10.1371/journal.pone.0333494 (PMC12533894; doi:10.1371/journal.pone.0333494)
Supplement: Attachment — Submitted filename: PONE-D-23-22373_comnent.pdf [file pone.0333494.s010.pdf]

# PLOS ONE

## Long-term pre-exposure prophylaxis retention among men who have sex with men and transgender persons: systematic review and meta-analysis

--Manuscript Draft--

| | |
|---|---|
| **Manuscript Number:** | PONE-D-23-22373 |
| **Article Type:** | Research Article |
| **Full Title:** | Long-term pre-exposure prophylaxis retention among men who have sex with men and transgender persons: systematic review and meta-analysis |
| **Short Title:** | Long-term PrEP retention among MSM and TGP |
| **Corresponding Author:** | Feline de la Court, MSc<br>Public Health Service of Amsterdam: GGD Amsterdam<br>Amsterdam, NETHERLANDS |
| **Keywords:** | PrEP; HIV; MSM; TGP |
| **Abstract:** | Successful implementation of pre-exposure prophylaxis (PrEP) requires long-term retention in PrEP care among those with increased likelihood of HIV. This study aimed to estimate the proportion of long-term retention in PrEP care and extent to which variability in PrEP retention is attributable to population- and program-specific characteristics among men who have sex with men (MSM) and transgender persons (TGP). We performed a systematic review and meta-analysis examining PrEP studies and conference abstracts retrieved from the PubMed and Ovid online databases, capturing demonstration projects or observational studies published from January 1, 2010 to March 24, 202[illegible]e included 84 studies (totaling 90 study "cohorts" analyzed) that reported on retenti[illegible]f oral PrEP for HIV and included predominantly MSM and TGP. Retention in PrEP care (i.e., proportion of participants continuing PrEP) was obtained from each study and used to estimate the cumulative probability of remaining on PrEP [illegible]d rate of PrEP discontinuation over time via a random-effects meta-analysis survival [illegible]del. We examined sources of heterogeneity by including study-level covariates in this model. The pooled cumulative probability of PrEP retention was 77.0%, 64.7%, 48.5%, and 24.1% at 6, 12, 24, and 60 months, respectively. Rates of PrEP discontinuation were significantly (p<0.05) lower in studies from Europe, Australia and multiple regions (vs. North America), and with ≥3-monthly follow-up (vs. <3). Rates were significantly higher in studies from Africa (vs. North America), with lower median age, higher proportions of non-MSM/TGP participants, higher proportions of participants with unspecified (vs. mixed) ethnicity, with unspecified (vs. daily) PrEP regimen, free-of-charge (vs. unspecified) STI testing, whose data were from conference abstracts (vs. peer-reviewed papers), and that were more recent. PrEP retention decreased over time and differed across population and program-specific characteristics. The heterogeneity across studies highlights PrEP implementation challenges and need for tailored retention strategies. |
| **Order of Authors:** | Feline de la Court, MSc |
| | Maria Prins, PhD |
| | Elske Hoornenborg, PhD |
| | Maarten F. Schim Van De Loeff, PhD |
| | Liza Coyer, PhD |
| | Anders Boyd, PhD |
| **Opposed Reviewers:** | |
| **Additional Information:** | |
| **Question** | **Response** |
| **Financial Disclosure**<br><br>Enter a financial disclosure statement that | MP obtained unrestricted research grants and speaker/ advisory fees from Gilead Sciences, Abbvie and MSD; all of which were paid to her institute and were unrelated to the current work. EH obtained unrestricted research grants from Gilead Sciences, |

| | |
|---|---|
| describes the sources of funding for the work included in this submission. Review the submission guidelines for detailed requirements. View published research articles from *PLOS ONE* for specific examples.<br><br>This statement is required for submission and **will appear in the published article** if the submission is accepted. Please make sure it is accurate.<br><br>**Unfunded studies**<br>Enter: *The author(s) received no specific funding for this work.*<br><br>**Funded studies**<br>Enter a statement with the following details:<br>• Initials of the authors who received each award<br>• Grant numbers awarded to each author<br>• The full name of each funder<br>• URL of each funder website<br>• Did the sponsors or funders play any role in the study design, data collection and analysis, decision to publish, or preparation of the manuscript?<br>• **NO** - Include this sentence at the end of your statement: *The funders had no role in study design, data collection and analysis, decision to publish, or preparation of the manuscript.*<br>• **YES** - Specify the role(s) played.<br><br>* typeset | which were paid to her institute and were unrelated to the current work. The institution of M. F. Schim van der Loeff receives study funding from GSK; he served on advisory boards of GSK and Merck/MSD; fees were paid to his institution. The other authors report no conflicts of interest.<br><br>The current study is part of the OptiPrEP (optimizing pre-exposure prophylaxis roll-out among men having sex with men) project, funded by the Aidsfonds (P-54601).<br><br>The funders had no role in study design, data collection and analysis, decision to publish, or preparation of the manuscript. |
| **Competing Interests**<br><br>Use the instructions below to enter a competing interest statement for this submission. On behalf of all authors, disclose any competing interests that could be perceived to bias this work—acknowledging all financial support and any other relevant financial or non-financial competing interests.<br><br>This statement is required for submission and **will appear in the published article** if the submission is accepted. Please make sure it is accurate and that any funding | MP obtained unrestricted research grants and speaker/ advisory fees from Gilead Sciences, Abbvie and MSD; all of which were paid to her institute and were unrelated to the current work. EH obtained unrestricted research grants from Gilead Sciences, which were paid to her institute and were unrelated to the current work. The institution of M. F. Schim van der Loeff receives study funding from GSK; he served on advisory boards of GSK and Merck/MSD; fees were paid to his institution.<br><br>The authors have declared that no competing interests exist |

| | |
|---|---|
| sources listed in your Funding Information later in the submission form are also declared in your Financial Disclosure statement.<br><br>View published research articles from *PLOS ONE* for specific examples.<br><br>**NO authors have competing interests**<br><br>Enter: *The authors have declared that no competing interests exist.*<br><br>**Authors with competing interests**<br><br>Enter competing interest details beginning with this statement:<br><br>*I have read the journal's policy and the authors of this manuscript have the following competing interests: [insert competing interests here]*<br><br>* typeset | |
| **Ethics Statement**<br><br>Enter an ethics statement for this submission. This statement is required if the study involved:<br><br>• Human participants<br>• Human specimens or tissue<br>• Vertebrate animals or cephalopods<br>• Vertebrate embryos or tissues<br>• Field research<br><br>Write "N/A" if the submission does not require an ethics statement.<br><br>General guidance is provided below. Consult the submission guidelines for detailed instructions. **Make sure that all information entered here is included in the Methods section of the manuscript.** | N/A |

| | |
|---|---|
| **Format for specific study types**<br><br>**Human Subject Research (involving human participants and/or tissue)**<br>• Give the name of the institutional review board or ethics committee that approved the study<br>• Include the approval number and/or a statement indicating approval of this research<br>• Indicate the form of consent obtained (written/oral) or the reason that consent was not obtained (e.g. the data were analyzed anonymously)<br><br>**Animal Research (involving vertebrate animals, embryos or tissues)**<br>• Provide the name of the Institutional Animal Care and Use Committee (IACUC) or other relevant ethics board that reviewed the study protocol, and indicate whether they approved this research or granted a formal waiver of ethical approval<br>• Include an approval number if one was obtained<br>• If the study involved *non-human primates*, add *additional details* about animal welfare and steps taken to ameliorate suffering<br>• If anesthesia, euthanasia, or any kind of animal sacrifice is part of the study, include briefly which substances and/or methods were applied<br><br>**Field Research**<br><br>Include the following details if this study involves the collection of plant, animal, or other materials from a natural setting:<br>• Field permit number<br>• Name of the institution or relevant body that granted permission | |
| **Data Availability**<br><br>Authors are required to make all data underlying the findings described fully available, without restriction, and from the time of publication. PLOS allows rare exceptions to address legal and ethical concerns. See the PLOS Data Policy and FAQ for detailed information. | Yes - all data are fully available without restriction |

| | |
|---|---|
| A Data Availability Statement describing where the data can be found is required at submission. Your answers to this question constitute the Data Availability Statement and **will be published in the article**, if accepted.<br><br>**Important:** Stating 'data available on request from the author' is not sufficient. If your data are only available upon request, select 'No' for the first question and explain your exceptional situation in the text box.<br><br>Do the authors confirm that all data underlying the findings described in their manuscript are fully available without restriction? | |
| **Describe where the data may be found in full sentences. If you are copying our sample text, replace any instances of XXX with the appropriate details.**<br><br>• If the data are **held or will be held in a public repository**, include URLs, accession numbers or DOIs. If this information will only be available after acceptance, indicate this by ticking the box below. For example: *All XXX files are available from the XXX database (accession number(s) XXX, XXX.*).<br>• If the data are all contained **within the manuscript and/or Supporting Information files**, enter the following: *All relevant data are within the manuscript and its Supporting Information files.*<br>• If neither of these applies but you are able to provide **details of access elsewhere**, with or without limitations, please do so. For example:<br><br>*Data cannot be shared publicly because of [XXX]. Data are available from the XXX Institutional Data Access / Ethics Committee (contact via XXX) for researchers who meet the criteria for access to confidential data.*<br><br>*The data underlying the results presented in the study are available from (include the name of the third party* | All data and analyses used in this study are available on GitHub. |

| | |
|---|---|
| *and contact information or URL).*<br>• This text is appropriate if the data are owned by a third party and authors do not have permission to share the data.<br><br>* typeset | |
| Additional data availability information: | |

Powered by Editorial Manager® and ProduXion Manager® from Aries Systems Corporation

*Amsterdam, July 17, 2023*

**From:** Feline de la Court, MSc
Department of Infectious Diseases, Research and Prevention
Public Health Service of Amsterdam
Nieuwe Achtergracht 100, 1018 WT Amsterdam, the Netherlands
E-mail: fdlcourt@ggd.amsterdam.nl; telephone: +31 (0)6 37 42 35 06

**To:** Prof. Matthew J. Mimiaga
Editor at *PLOS ONE*
Brown University
United States

Dear Prof. Matthew J. Mimiaga,

We are pleased to submit, for your appreciation, our research article entitled "**Long-term pre-exposure prophylaxis retention among men who have sex with men and transgender persons: systematic review and meta-analysis**" for publication as an original article in *PLOS One*.

Pre-exposure prophylaxis (PrEP) is a highly effective prevention strategy against HIV, yet its successful implementation requires long-term retention in PrEP care among those with higher likelihood of acquiring HIV. Men who have sex with men (MSM) and transgender persons (TGP) are among the most important populations for PrEP use considering their increased behavioral susceptibility for HIV. However, in these groups, the proportion of individuals retained in PrEP care is currently suboptimal, especially in the long term. This study therefore performed a systematic review and meta-analysis to estimate the proportion of long-term retention in PrEP care in studies that predominately included MSM and TGP. We also analyzed the extent to which variability in PrEP retention is attributable to population- and to program-specific characteristics.

Our study is unique in providing insight on long-term retention up to 60 months across 90 study cohorts and findings contribute to strategies for effective and sustained PrEP care. We estimated a 77.0% cumulative probability of remaining on PrEP at 6 months after PrEP initiation. This probability declined to 48.5% at 24 months and to 24.1% at 60 months. Along with the substantial heterogeneity in this probability observed across studies, and the multiple factors associated with discontinuation, our findings highlight that continuing PrEP use is challenging for the majority of those who start PrEP. We conclude that PrEP retention became markedly lower with longer time on PrEP and differed between populations and across program-specific characteristics for which PrEP retention strategies should be tailored.

Given that these findings could help improve prevention of HIV through PrEP, we feel that *PLOS ONE* would be aa very suitable journal to openly disseminate these results. We suggest Matthew J. Mimiaga as an appropriate Academic Editor to handle our manuscript due to their expertise in HIV and PrEP.

We hereby inform you that this paper has not been submitted or published elsewhere. Moreover, all listed authors have participated meaningfully in the study, approved its content and carried full responsibility for writing this paper.

We thank you for your consideration and greatly look forward to your response.

Sincerely,
On behalf of the authors,

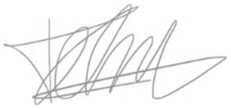

Feline de la Court

Click here to access/download;Manuscript;Manuscript.docx

# Long-term pre-exposure prophylaxis retention among men who have sex with men and transgender persons: systematic review and meta-analysis

Feline de la Court[1]*, Maria Prins[1,3], Elske Hoornenborg[1], Maarten F. Schim Van De Loeff[1, 3], Liza Coyer[1,3] ¶, Anders Boyd[1,2] ¶

[1] Department of Infectious Diseases, Public Health Service of Amsterdam, Amsterdam, the Netherlands;

[2] Stichting hiv monitoring, Amsterdam, the Netherlands;

[3] Department of Infectious Diseases, Amsterdam institute for Infection & Immunity (AII), Amsterdam UMC, University of Amsterdam, Amsterdam, the Netherlands.

* Corresponding author:

E-mail: fdlcourt@ggd.amsterdam.nl (F.d.l.C.)

¶These authors contributed equally to this work.

# Abstract

Successful implementation of pre-exposure prophylaxis (PrEP) requires long-term retention in PrEP care among those with increased likelihood of HIV. This study aimed to estimate the proportion of long-term retention in PrEP care and extent to which variability in PrEP retention is attributable to population- and program-specific characteristics among men who have sex with men (MSM) and transgender persons (TGP). We performed a systematic review and meta-analysis examining PrEP studies and conference abstracts retrieved from the PubMed and Ovid online databases, capturing demonstration projects or observational studies published from January 1, 2010 to March 24, 2021. We included 84 studies (totaling 90 study "cohorts" analyzed) that reported on retention of oral PrEP for HIV and included predominantly MSM and TGP. Retention in PrEP care (i.e., proportion of participants continuing PrEP) was obtained from each study and used to estimate the cumulative probability of remaining on PrEP and rate of PrEP discontinuation over time via a random-effects meta-analysis survival model. We examined sources of heterogeneity by including study-level covariates in this model. The pooled cumulative probability of PrEP retention was 77.0%, 64.7%, 48.5%, and 24.1% at 6, 12, 24, and 60 months, respectively. Rates of PrEP discontinuation were significantly ($p<0.05$) lower in studies from Europe, Australia and multiple regions (vs. North America), and with ≥3-monthly follow-up (vs. <3). Rates were significantly higher in studies from Africa (vs. North America), with lower median age, higher proportions of non-MSM/TGP participants, higher proportions of participants with unspecified (vs. mixed) ethnicity, with unspecified (vs. daily) PrEP regimen, free-of-charge (vs. unspecified) STI testing, whose data were from conference abstracts (vs. peer-reviewed papers), and that were more recent. PrEP retention decreased over time and differed across population and program-specific characteristics. The heterogeneity across studies highlights PrEP implementation challenges and need for tailored retention strategies.

# Introduction

Pre-exposure prophylaxis (PrEP) is a highly effective prevention strategy against HIV, yet its successful implementation requires long-term retention in PrEP care among those with higher likelihood of acquiring HIV[1]. Long-term retention refers to the sustained engagement in PrEP care when one's likelihood of HIV infection is increased, which is a reflection of prevention-effective adherence [2]. Men who have sex with men (MSM) and transgender persons (TGP) are an important target population for PrEP considering the increased HIV prevalence among these populations [3]. However, in these groups the proportion of individuals retained in PrEP care is currently suboptimal, especially in the long term[4].

Given that the incidence of HIV is particularly high among those who discontinued or had gaps in their PrEP use[5, 6], low long-term retention in PrEP care is concerning[5]. A previous systematic review on PrEP retention among MSM and TGP has shown a pooled proportion of PrEP discontinuation of 32% at 6 months and 35% up to 12 months[7]. That study evaluated 59 studies, but data around and beyond 12 months of follow-up were scarce. Furthermore, research has suggested that retention is often lower among MSM and TGP who are adolescent or in early-adulthood, socioeconomically disadvantaged or from ethnic minority groups[5, 8]. Retention outcomes may also be attributable to factors related to location, financing and implementation or research in PrEP care[9-11]. So far, no previous review study has provided an aggregated analysis on the extent to which such program-specific characteristics affect PrEP retention outcomes.

This systematic review and meta-analysis estimated the proportion of long-term retention in PrEP care in studies that predominately included MSM and TGP. We also analyzed

the extent to which variability in PrEP discontinuation is attributable to population- and to program-specific characteristics. Such insights may contribute to strategies for effective and sustained PrEP care.

# Methods

## Study design

We performed this systematic review and meta-analysis in accordance with guidelines from the Cochrane Handbook for Systematic Reviews of Interventions[12] and described this analysis using the Preferred Reporting Items for Systematic Review and Meta-analysis guidelines[13] (S1 PRISMA checklist). This study was not registered in a register for systematic reviews.

## Search strategy and inclusion criteria: oral PrEP continuation, measurement and reporting.

A systematic search for peer-reviewed papers and conference abstracts was conducted using the PubMed and Ovid online databases (but not Embase due to access restriction), retrieving studies published from January 1, 2010 to March 24, 2021. The full search strategy is given in S1 Table. Additional abstracts were retrieved from conference databases of the International AIDS Conference and the Conference on Retroviruses and Opportunistic Infections from 2017-2021. We used EndNote X9.3.3 to manage all citations.

An initial selection was made based on title and abstract, and two independent reviewers (F.d.l.C. and L.C.) conducted a full-text screening for study inclusion. We included only studies that reported on retention of people using oral PrEP for HIV (i.e., fixed dose

combination of emtricitabine and tenofovir disoproxil fumarate) taken daily, event-driven or both, and that were conducted among a predominantly (≥60%) MSM and/or TGP population. Moreover, studies were included if follow-up time and proportion of participants retained in care from PrEP initiation to discontinuation were reported or could be calculated for at least one of the pre-defined follow-up time points (i.e., beginning at month 3 since PrEP initiation, and continuing for every 3 months until month (60). Eligible study designs were: unblinded randomized controlled trials (RCT), open label extension (OLE) studies, longitudinal observational cohort studies, demonstration projects, implementation projects, and studies conducted in primary care or other routine public health settings. Double- and single-blinded RCTs were excluded to ensure that the outcomes approximated real-world settings. Studies were also excluded if follow-up parameters were unclear (i.e., follow-up did not begin at start of PrEP use or only median follow-up time was reported) or not mentioned. No restrictions were imposed based on language or geographical location. If multiple studies described the same study population or cohort, the study with the longest follow-up time and most complete follow-up information was included. For open-label RCTs containing both an "intervention" and "standard of care" arm, each arm was included as a separate study cohort.

## Outcome

The primary outcome was retention in PrEP care, defined as the proportion of participants reported to be using PrEP at each of the pre-defined time points (every 3 months from month 3 to month 60). This proportion was calculated by dividing the number of participants reported to be on PrEP by the total number of participants who initiated PrEP in the study.

## Data extraction

Data from all included studies were extracted by two reviewers (F.d.l.C. and L.C.) independently using a standardized form. Consensus was reached for any discrepancies in the data extracted between reviewers. The target population was listed as MSM if the proportion of MSM was ≥80% in the study population.

Study variables further included: region (i.e., North America, Europe, Australia, South America, Africa, Asia, or multiple); calendar years at the beginning, completion, and publication of study; study population (i.e., MSM [when >80% is MSM] or "MSM, TGP and other"); percentage of MSM, TGP, or other in the study population; geographic setting (i.e., urban, rural, or mixed); ethnicity of study population (i.e., mixed, Black only, Asian only, unspecified); median age; frequency of follow-up visits (i.e., every 3 months or more frequently, or less frequently than every 3 months); outcome definition (i.e., discontinuation, loss-to-follow-up, retention or mixed/other); publication type (i.e., paper or conference abstract); PrEP regimen (i.e., daily, event-driven, both, or unspecified); PrEP and STI testing costs for the participant (i.e., completely free-of-charge, not completely free-of-charge, or unspecified). We included the "unspecified" category to assess missing or unclear results in the synthesis of the last two named variables.

## Risk of bias assessment

The methodological quality and risk of bias of included studies was assessed by one reviewer (F.d.l.C.) using the Newcastle-Ottawa Quality Assessment Scale for cohort studies. Since we analyzed RCTs as cohort studies, these too have been evaluated with the Newcastle-Ottawa Quality Assessment Scale for cohort studies rather than an RCT-specific tool. The risk of bias assessment has been summarized in S2 Table.

## Statistical analysis

We used the proportion of participants reporting to be using PrEP to model the cumulative probability of remaining on PrEP over time. This probability could be estimated for an individual study *i*, defined as the survival probability, $S_i$, using a random-effects model similar to that described in Arends et al.[14]. Reported survival probabilities were transformed as $\ln(-\ln(S_i))$ and were regressed on a fixed-effect parameter including time and a vector of random coefficients and its corresponding design matrix. This design matrix contained an intercept and time effect.

The error term was modeled as a column vector of residuals containing a covariance matrix, V, whose main diagonal was set equal to the squared standard errors of the survival probabilities. These standard errors were calculated using Greenwood's formula and were made to correspond to the ln(-ln) survival probabilities using the formula in Arends et al.[14]. Survival probabilities from the same study can be considered correlated, whose covariance is iteratively estimated as blocks of within-study covariance matrices within the covariance matrix, V. However, the model failed to achieve convergence and we were thus unable to account for within-study correlation.

The model was used to generate the predicted cumulative probability of remaining on PrEP and its 95% confidence interval (CI). We estimated the model using the "meglm" command in STATA. To assess the sources of heterogeneity, we could estimate the proportion of PrEP retention over time using the same random-effects survival model and compare proportions across levels of study characteristics by including additional covariates in the fixed-effect parameter of the model. The resulting parameter estimate is a hazards ratio (HR)

and 95%CI comparing the rates of PrEP discontinuation across levels of study variables. This analysis is referred herein to as meta-regression.

As several diagnostic statistics have not yet been developed for the random-effects survival model approach, we employed a meta-analysis random-effects model with between-study variance calculated using restricted maximum likelihood estimation[15]. This model was run for each of the timepoints at which the majority of studies reported data (i.e., months 3, 6, 9, and 12). We used the “rma” function of the “metafor” package in R to estimate the model. From this model, we calculated the measure of heterogeneity $I^2$ and tested the null hypothesis of no between-study heterogeneity using the Q statistic at each timepoint. We assessed the influence of outlying studies at each timepoint using the method developed by Viechtbauer and Cheung[16] in the “metafor” package in R. We also assessed for publication bias at these same timepoints using the Egger test of bias[17] with $p<0.05$ indicating significant publication bias.

We carried out analysis using STATA (v15.1, College Station, TX, USA) and R (v4.0.3, Vienna, Austria). We defined significance as $p<0.05$.

# Results

## Characteristics of included studies

Fig 1 presents the flow-diagram for study selection. The database search identified 6853 studies published between 2010 and 2021. After removal of duplicates (n=3627) and exclusion based on title and abstract (n=2760), 466 full-text studies were screened. From these, 382 studies were excluded for reasons which are summarized in Fig 1. Finally, 84 studies were selected (S1 References). Of these, 6 were open-label RCTs containing both an “intervention”

and “standard of care” arm; each of these arms were considered as separate study cohorts and hence 90 study cohorts were included in the analysis.

**Fig 1. Flow diagram of study search and inclusion.** *Studies with two study arms (n=6) were included as one study but were analyzed separately; in total, the number of inclusions is therefore 84 individual studies but this amounts to 90 inclusions (i.e., study cohorts) in the analyses. Abbreviations: MSM=men who have sex with men; TGP=transgender person; PrEP=pre-exposure prophylaxis.

The 90 study cohorts were reported in 53 peer-reviewed papers and 31 conference abstracts. The largest proportion were cohort studies (n=41; 45.6%), followed by demonstration or implementation projects (n=29; 32.2%). The majority of studies were conducted in the USA or Canada (n=50; 55.6%), Asian countries (n=15; 16.7%) or European countries (n=10; 11.1%), most studies reported on a population of individuals with mixed ethnicity (n=53; 58.9%) and in urban settings (n=83; 92.2%). Characteristics of all the included studies have been summarized in Table 1 and individually described in S3 Table and per region in S4 Table.

**Table 1. Description of study characteristics.**

| **Variables** | **Number of studies**<br>*n/N (%) or median [IQR]* |
|---|---|
| **Region** | |
| North America | 53/90 (58.9) |
| Europe | 5/90 (5.6) |
| Australia | 6/90 (6.7) |
| South America | 6/90 (6.7) |
| Africa | 5/90 (5.6) |
| Asia | 12/90 (13.3) |
| Multiple | 3/90 (3.3) |
| **Calendar year** | |

| | |
|---|---|
| Year begun * | 2015 [2014-2017] |
| Year completed † | 2017 [2016-2018] |
| Year published | 2019 [2018-2020] |
| **Study population** | |
| MSM (>80%) | 72/90 (80.0) |
| MSM, TGP and other | 18/90 (20.0) |
| **Percentage of study population (%)** ‡ | |
| MSM | 98.5 [91.7-100] |
| TGP | 4.4 [1.0-9.8] |
| Other | 5.0 [1.3-10.5] |
| **Geographic setting** | |
| Urban | 83/90 (92.2) |
| Urban/rural or rural only | 7/90 (7.8) |
| **Ethnicity** | |
| Mixed | 53/90 (58.9) |
| Black only | 6/90 (6.7) |
| Asian only | 3/90 (3.3) |
| Unspecified | 28/90 (31.1) |
| **Median of study median age (years)** ‖ | 32 [25-35] |
| **Frequency of follow-up visits** | |
| <3 months | 72/87 (82.8) |
| ≥3 months | 15/87 (17.2) |
| **Retention terminology**¶ | |
| Discontinuation | 16/89 (18.0) |
| Follow-up | 16/89 (18.0) |
| Mixed/Other | 17/89 (19.1) |
| Retention | 40/89 (44.9) |
| **Publication type** | |
| Paper | 58/90 (64.4) |
| Abstract | 32/90 (35.6) |
| **PrEP regimen** | |
| Daily | 47/90 (52.2) |
| Event-Driven | 1/90 (1.1) |
| Both | 6/90 (6.7) |
| Unspecified | 36/90 (40.0) |
| **PrEP cost** | |
| Completely free | 25/90 (27.8) |
| Not entirely free | 10/90 (11.1) |
| Unspecified | 55/90 (61.1) |
| **STI testing cost** | |
| Completely free | 1/90 (1.1) |
| Not entirely free | 6/90 (6.7) |
| Unspecified | 83/90 (92.2) |

Data retrieved from 84 studies published between 2010 and 2021. All continuous variables are summarized as median (IQR). Abbreviations: IQR= interquartile range; MSM=men who have sex with men; TGP=transgender person; PrEP=pre-exposure prophylaxis; STI=sexually transmitted infection. *Defined as the year in which the study with PrEP follow-up was

initiated; data missing for 8 studies. [†]Defined as the year in which the study with PrEP follow-up was stopped; data missing for 8 studies. [‡]Missing data or nonapplicable study population for MSM in 9 studies, for TGP in 46 studies and for other populations in 65 studies. [‖]Missing data in 56 studies. [¶]Defined as the terminology used in the study to indicate or determine PrEP retention.

## Long-term PrEP retention

S5 Table displays retention outcomes for all included studies per pre-defined time point. Fig 2 depicts the observed cumulative probability of remaining on PrEP for each study and timepoint, alongside the predicted probability from the random-effects survival model. The model tends to give lower estimates of this probability than observed, particularly at later timepoints. From the model, the pooled cumulative probability of remaining on PrEP was 77.0% (95%CI=73.4-80.2) at 6 months, 64.7% (95%CI=57.6-70.9%) at 12 months, 48.5% (95%CI=36.4-59.4) at 24 months, and 24.1% (95%CI=10.3-41.2) at 60 months.

**Fig 2. Cumulative probability of individuals remaining on pre-exposure prophylaxis for preventing HIV, over time.** Data retrieved from 90 studies conducted between 2010 and 2020 and ranging from 3 to 60 months of follow-up (median=12). Individual values for studies are depicted as grey dots.

## Determinants of variability in discontinuation

As shown in Table 2, results of the meta-regression analysis revealed significantly lower rates of PrEP discontinuation in studies conducted in Europe ($p$<0.001), Australia ($p$<0.001) and multiple regions ($p$=0.003) compared to North America, and in studies with follow-up intervals of more than three months ($p$=0.028) compared to those with follow-up

visits every 3 months or less. In contrast, significantly higher rates of PrEP discontinuation were observed in studies conducted in Africa (vs. North America; $p$=0.005), studies with unspecified ethnicity (vs. mixed ethnicity; $p$=0.031), unspecified PrEP regimen (vs. daily; $p$<0.001), free-of-charge STI testing (vs. unspecified; $p$=0.007) and in cohorts reported in abstracts (vs. peer-reviewed papers; $p$<0.001). PrEP discontinuation was found to be significantly higher with more recent study start dates ($p$=0.015), and with larger proportions of participants other than MSM or TGP ($p$=0.029); PrEP discontinuation was significantly lower as median age of participants was lower ($p$=0.035). There was no evidence that outcome definition, either urban or rural geographic setting, or PrEP cost were associated with differences in PrEP discontinuation. Multivariable analysis was precluded by the fact that certain levels of factors were commonly shared within studies.

**Table 2. Meta-regression for relative differences in discontinuing PrEP for preventing HIV.**

| Variables | Univariable model | | |
|---|---|---|---|
| | *HR* | *(95% CI)* | *P* |
| **Region** | | | |
| North America | Ref | | |
| Europe | 0.31 | (0.15-0.62) | 0.001 |
| Australia | 0.30 | (0.17-0.56) | <0.001 |
| South America | 0.95 | (0.54-1.68) | 0.87 |
| Africa | 2.47 | (1.32-4.62) | 0.005 |
| Asia | 1.00 | (0.64-1.58) | 0.99 |
| Multiple | 0.36 | (0.18-0.71) | 0.003 |
| **Calendar year (per year)** | | | |
| Year begun * | 1.11 | (1.02-1.20) | 0.015 |
| Year completed † | 1.08 | (0.98-1.20) | 0.13 |
| Year published | 1.11 | (0.98-1.27) | 0.10 |
| **Study population** | | | |
| MSM (>80%) | Ref | | |
| MSM, TGP and other | 1.14 | (0.78-1.66) | 0.51 |
| **Percentage of study population (per %)** ‡ | | | |
| MSM | 0.98 | (0.97-1.00) | 0.053 |
| TGP | 1.02 | (0.99-1.04) | 0.11 |
| Other | 1.03 | (1.00-1.06) | 0.029 |

| | | | |
|---|---|---|---|
| **Geographic setting** | | | |
| Urban | Ref | | |
| Urban and rural or rural only | 1.15 | (0.69-1.91) | 0.60 |
| **Ethnicity** | | | |
| Mixed | Ref | | |
| Black only | 0.84 | (0.45-1.57) | 0.59 |
| Asian only | 0.85 | (0.28-3.60) | 0.77 |
| Unspecified | 1.47 | (1.04-2.08) | 0.031 |
| **Median of study median age (per year)** ‖ | 0.96 | (0.92-0.99) | 0.035 |
| **Frequency of follow-up visits** | | | |
| <3 months | Ref | | |
| ≥3 months | 0.58 | (0.36-0.94) | 0.028 |
| **Outcome definition** ¶ | | | |
| Discontinuation | Ref | | |
| Follow-up | 0.99 | (0.58-1.69) | 0.97 |
| Mixed/Other | 0.65 | (0.39-1.07) | 0.091 |
| Retention | 1.09 | (0.71-1.67) | 0.71 |
| **Study type** | | | |
| Paper | Ref | | |
| Abstract | 1.94 | (1.40-2.67) | <0.001 |
| **PrEP regimen** | | | |
| Daily | Ref | | |
| Event-Driven | 0.35 | (0.10-1.27) | 0.11 |
| Both | 0.95 | (0.53-1.71) | 0.86 |
| Unspecified | 2.06 | (1.53-2.79) | <0.001 |
| **PrEP cost** | | | |
| Completely free | Ref | | |
| Not entirely free | 1.50 | (0.78-2.87) | 0.23 |
| Unspecified | 1.51 | (0.97-2.36) | 0.070 |
| **STI cost** | | | |
| Completely free | 4.45 | (1.50-13.17) | 0.007 |
| Not entirely free | 0.93 | (0.51-1.68) | 0.80 |
| Unspecified | Ref | | |

Meta-regression performed on data retrieved from 90 study cohorts conducted between 2010 and 2021. This model was run for each of the timepoints at which the majority of studies reported data (i.e., months 3, 6, 9, and 12). Abbreviations: MSM=men who have sex with men; TGP=transgender person; PrEP=pre-exposure prophylaxis; STI=sexually transmitted infection. *Defined as the year in which the study with PrEP follow-up was initiated; data missing for 8 studies. †Defined as the year in which the study with PrEP follow-up was stopped; data missing for 8 studies. ‡Missing data or nonapplicable study population in 9 (MSM), 46

(TGP) and 65 (other) studies. [|]Missing data in 56 studies. [¶]Defined as the terminology used in the study to indicate or determine PrEP retention.

## Quality assessment

The risk of bias assessment shows that there was a low overall risk of bias (S2 Table). Heterogeneity was consistently high between-studies from data reported throughout the first year of follow-up (month 3, $I^2$=99.2%; month 6, $I^2$=99.3%; month 9, $I^2$=99.3%; month 12, $I^2$=99.6%). Accordingly, between-study heterogeneity was significant ($p<0.0001$ for all time points). There were no influential studies identified at any of the timepoints (data not shown). Also, significant publication bias was observed at each of these timepoints (month 3, $p<0.0001$; month 6, $p<0.0001$; month 9, $p=0.0005$; month 12, $p<0.0001$; funnel plots provided in S1 Fig), with larger studies showing higher cumulative probabilities of remaining on PrEP.

# Discussion

This systematic review and meta-analysis estimated a 77.0% cumulative probability of remaining on PrEP at 6 months after PrEP initiation. This probability declined to 48.5% at 24 months and to 24.1% at 60 months. Along with the substantial heterogeneity in this probability observed across studies, and the multiple factors associated with discontinuation, our findings highlight that continuing PrEP use is challenging for the majority of those who start PrEP.

It should be noted that PrEP discontinuation is not *per se* an undesirable endpoint. If individuals have a low likelihood or low perceived likelihood of acquiring HIV, discontinuation is warranted with appropriate counselling[18]. Research shows that those who discontinue PrEP when chances of acquiring HIV are low, may be more inclined to re-initiate

when the likelihood of HIV increases again[19, 20]. Hence, efforts to improve PrEP retention should focus on the groups discontinuing PrEP while still substantially vulnerable for HIV. That being said, re-initiation with increased HIV likelihood implies timely access to PrEP, which is not always the case, and counselling after discontinuation may also lead to HIV prevention modes other than PrEP; highlighting the importance of tailored and well-timed PrEP counselling. It is also important to consider that the definitions used in studies may also shape the way discontinuation is addressed. Our findings show that studies who use the term ‘retention’ rather than ‘discontinuation’ had higher rates of PrEP discontinuation (albeit non-significantly). Further research comparing the ways in which programs encourage retention, or conversely, avoid discontinuation, and how definitions shape outcomes could clarify this finding.

Although MSM and TGP are key target populations for PrEP use, other populations (e.g., women, heterosexuals, people who inject drugs) engaging in behaviors and/or networks that increase the likelihood of HIV, are less likely to be on PrEP, regardless of need[21]. Even though we included studies that were predominately MSM and TGP, those including a larger proportion of participants outside these groups, most of which were unspecified, had higher rates of PrEP discontinuation. This may be attributable to PrEP care not being tailored to non-MSM groups[7, 22], and may be related to varying levels of perceived likelihood of acquiring HIV, which do not always match the individual’s behavioral vulnerability to HIV[23]. There was a lot of missing information concerning population composition across studies, making it difficult to attribute the lower retention levels to a specific subgroup.

Another population-specific factor related to PrEP discontinuation was age. We found that with older age, the rates of PrEP discontinuation were lower. This finding mirrors other

outcomes involved in PrEP use, such as more frequent switching between daily and event-driven regimens[24] and poorer adherence[25] more commonly found in younger individuals. Moreover, younger MSM have been found to often experience barriers to PrEP care related to access, costs, stigma, and perceived lower likelihood of HIV despite behavioral vulnerability[26], which could reflect the lower levels of PrEP retention found among younger MSM[27, 28].

Furthermore, there were regional differences in PrEP discontinuation rates. When compared to North America, studies in Europe and Australia had lower PrEP discontinuation rates, while those in Africa had higher rates. Regional differences are difficult to explain, but could be due to variations in levels of PrEP availability or the ability of healthcare systems to provide and support PrEP use and adherence. For instance, across Europe[29] and in Australia[30], multiple large-scale PrEP programs have been initiated, allowing for more affordable and easily accessible PrEP, while insurance-related and social stigma barriers observed in North America have been known to negatively impact PrEP use[21]. Moreover, research shows that low perceived likelihood of acquiring HIV, experienced stigma and difficulties in returning to PrEP care appointments, often experienced by MSM and TGP in African countries, could also hinder PrEP use[31-33]. The factors affecting PrEP use that were identified in these studies could have downstream consequences for PrEP retention, as evidenced in our meta-analysis. The observed regional differences could also be the result of differences in study populations and design; given that Asian studies included a lower proportion of MSM and more frequent visits were commonly found in North American, South American and African studies (S4 Table).

Rates of PrEP discontinuation were found to be significantly lower among studies with ≥3 monthly follow-up visits (vs. <3 monthly), suggesting that those who are required to engage in more intense PrEP care are more likely to discontinue PrEP use, which may be due to the experienced burden of frequent clinic visits[11] or changing sexual behavior over time. Earlier study start dates were also associated with lower rates of PrEP discontinuation. For these earlier studies (i.e., before 2016), which were more often demonstration or implementation projects, included individuals may have been more engaged and motivated to use PrEP, as formal PrEP access was limited[22, 34]. Balance is thus needed between the cons (e.g., burden of clinical visits, lower proportion of retention) and benefits (e.g., earlier diagnosis and treatment of STI and HIV) of more frequent PrEP visits.

One curious finding is the association between the unspecified levels of several factors and higher rates of PrEP discontinuation. For instance, PrEP discontinuation rates were higher when studies did not specify PrEP regimen (vs. daily regimen) and when studies did not specify ethnicity (vs. mixed ethnicity). Previous studies have shown racial disparities exist in PrEP retention[35]; as such, understanding what constitutes "unspecified" for these variables would appear valuable in identifying population- and program-specific factors influencing retention in PrEP care. However, large scale and long-term studies generally do not measure the impact of such factors on PrEP retention. This gap in knowledge could be due to the lack of collection of this data, as demonstrated in our meta-analysis. More consistency in the collection and reporting of information is needed when evaluating PrEP programs.

Strengths of our systematic review and meta-analysis are the large number of studies, long follow-up times, and the fact that we did not solely assess one aspect across studies (i.e., both population- and program-specific characteristics were studied). In addition, we included

studies reporting on TGP, an underrepresented population in research. This meta-analysis attempts to provide a much-needed summary of PrEP retention outcomes in the context of TGP groups; however, we acknowledge that this remains a prominent disparity in PrEP-related research.

There are nevertheless certain limitations of our study. First, publication bias was pervasive across studies; we therefore need to be aware of a potential overestimation of PrEP retention compared to routine practice[36]. Second, we only considered PrEP discontinuation as an outcome and assumed that those discontinuing PrEP did not re-initiate PrEP. A relatively large proportion of individuals are known to re-initiate PrEP after discontinuation[7], but the variation in definitions of PrEP discontinuation across studies makes it difficult to distinguish between reasons for discontinuing PrEP. Third, we did not search Embase due to access restrictions and did not include conferences such as the HIV Research for Prevention Conference; hence, although there is likely overlap across these databases/conferences, some studies may have been missed. Fourth, because certain levels of factors were commonly shared between studies, a multivariable meta-regression survival model could not be constructed. Also, the model tended to give lower estimates of the probability of remaining on PrEP than observed, particularly at later timepoints. Finally, this systematic review included a large number of studies, however a relatively limited number of studies were found to report long-term outcomes after month 24.

Our findings could assist in improving PrEP care as it is crucial that healthcare professionals engage and/or re-engage PrEP users in care when needed, by critically evaluating PrEP need and identifying barriers. Engagement in PrEP care should include informing and counseling by providing information on retention benefits or alternative methods for HIV

prevention, and the provision of multiple pathways back to PrEP care. Moreover, our findings might warrant tailored PrEP care (e.g., less frequent visits, free-of-charge PrEP and STI testing, or online PrEP care), considering the variability in PrEP retention across different population characteristics, such as ethnicity, age and being non-MSM/TGP. We must be aware that different populations may experience different barriers to PrEP care, which could lead to discontinuation in varying ways.

In conclusion, PrEP retention became markedly lower with longer time on PrEP (i.e., follow-up) and differed between populations and across program-specific characteristics. Studies must provide consistent information on discontinuing PrEP care for clear comparisons across studies, which could provide a more precise understanding of the structural and individual factors influencing the variability in PrEP retention. The heterogeneity found in the cumulative probability retained on PrEP across study cohort highlights the need for more tailored PrEP retention strategies.


# Acknowledgements

This study is part of the OptiPrEP (optimizing pre-exposure prophylaxis roll-out among men having sex with men) project, funded by the Aidsfonds (P-54601).


# Contributors

FdlC, LC, AB designed the study. FdlC and LC performed the data collection and AB performed the analyses. FdlC and AB drafted the manuscript, with the support of LC. All other authors contributed to the revision of the paper. All authors read and approved the final

manuscript. We also wish to acknowledge David O.T. ten Hoff for his contribution in earlier analyses of the study.

## Conflict of interest statement

MP obtained unrestricted research grants and speaker/ advisory fees from Gilead Sciences, Abbvie and MSD; all of which were paid to her institute and were unrelated to the current work. EH obtained unrestricted research grants from Gilead Sciences, which were paid to her institute and were unrelated to the current work. The institution of M. F. Schim van der Loeff receives study funding from GSK; he served on advisory boards of GSK and Merck/MSD; fees were paid to his institution. The other authors report no conflicts of interest.

## Funding statement

The current study is part of the OptiPrEP (optimizing pre-exposure prophylaxis roll-out among men having sex with men) project, funded by the Aidsfonds (P-54601).

## Data availability statement

All data and analyses used in this study are available on GitHub.

## References

1. Chou R, Evans C, Hoverman A, Sun C, Dana T, Bougatsos C, et al. Preexposure Prophylaxis for the Prevention of HIV Infection: Evidence Report and Systematic Review for the US Preventive Services Task Force. JAMA. 2019;321(22):2214-30.

2. Haberer JE, Bangsberg DR, Baeten JM, Curran K, Koechlin F, Amico KR, et al. Defining success with HIV pre-exposure prophylaxis: a prevention-effective adherence paradigm. Aids. 2015;29(11):1277-85.

3. Celum C, Baeten J. PrEP for HIV Prevention: Evidence, Global Scale-up, and Emerging Options. Cell Host & Microbe. 2020;27(4):502-6.

4. Stankevitz K, Grant H, Lloyd J, Gomez GB, Kripke K, Torjesen K, et al. Oral preexposure prophylaxis continuation, measurement and reporting. AIDS. 2020;34(12):1801-11.

5. Hojilla JC, Hurley LB, Marcus JL, Silverberg MJ, Skarbinski J, Satre DD, et al. Characterization of HIV Preexposure Prophylaxis Use Behaviors and HIV Incidence Among US Adults in an Integrated Health Care System. JAMA Netw Open. 2021;4(8):e2122692.

6. Spinelli MA, Laborde N, Kinley P, Whitacre R, Scott HM, Walker N, et al. Missed opportunities to prevent HIV infections among pre-exposure prophylaxis users: a population-based mixed methods study, San Francisco, United States. J Int AIDS Soc. 2020;23(4):e25472.

7. Zhang J, Li C, Xu J, Hu Z, Rutstein SE, Tucker JD, et al. Discontinuation, suboptimal adherence, and reinitiation of oral HIV pre-exposure prophylaxis: a global systematic review and meta-analysis. The Lancet HIV. 2022;9(4):e254-e68.

8. Caponi M, Burgess C, Leatherwood A, Molano LF. Demographic characteristics associated with the use of HIV pre-exposure prophylaxis (PrEP) in an urban, community health center. Prev Med Rep. 2019;15:100889-.

9. Krakower DS, Mayer KH. The role of healthcare providers in the roll out of preexposure prophylaxis. Curr Opin HIV AIDS. 2016;11(1):41-8.

10. Pinto RM, Lacombe-Duncan A, Kay ES, Berringer KR. Expanding Knowledge About Implementation of Pre-exposure Prophylaxis (PrEP): A Methodological Review. AIDS and Behavior. 2019;23(10):2761-78.

11. Whitfield THF, John SA, Rendina HJ, Grov C, Parsons JT. Why I Quit Pre-Exposure Prophylaxis (PrEP)? A Mixed-Method Study Exploring Reasons for PrEP Discontinuation and Potential Re-initiation Among Gay and Bisexual Men. AIDS and Behavior. 2018;22(11):3566-75.

12. Higgins JP, Thomas J, Chandler J, Cumpston M, Li T, Page MJ, et al. Cochrane handbook for systematic reviews of interventions: John Wiley & Sons; 2019.

13. Page MJ, McKenzie JE, Bossuyt PM, Boutron I, Hoffmann TC, Mulrow CD, et al. The PRISMA 2020 statement: an updated guideline for reporting systematic reviews. Systematic Reviews. 2021;10(1):89.

14. Arends LR, Hunink MG, Stijnen T. Meta-analysis of summary survival curve data. Stat Med. 2008;27(22):4381-96.

15. Viechtbauer W. Bias and Efficiency of Meta-Analytic Variance Estimators in the Random-Effects Model. Journal of Educational and Behavioral Statistics. 2005;30(3):261-93.

16. Viechtbauer W, Cheung MW. Outlier and influence diagnostics for meta-analysis. Res Synth Methods. 2010;1(2):112-25.

17. Egger M, Smith GD, Schneider M, Minder C. Bias in meta-analysis detected by a simple, graphical test. BMJ. 1997;315(7109):629.

18. Rutstein SE, Smith DK, Dalal S, Baggaley RC, Cohen MS. Initiation, discontinuation, and restarting HIV pre-exposure prophylaxis: ongoing implementation strategies. Lancet HIV. 2020;7(10):e721-e30.

19. Whitfield THF, John SA, Rendina HJ, Grov C, Parsons JT. Why I Quit Pre-Exposure Prophylaxis (PrEP)? A Mixed-Method Study Exploring Reasons for PrEP Discontinuation

and Potential Re-initiation Among Gay and Bisexual Men. AIDS Behav. 2018;22(11):3566-75.

20. Zucker J, Carnevale C, Richards P, Slowikowski J, Borsa A, Gottlieb F, et al. Predictors of Disengagement in Care for Individuals Receiving Pre-exposure Prophylaxis (PrEP). J Acquir Immune Defic Syndr. 2019;81(4):e104-e8.

21. Mayer KH, Agwu A, Malebranche D. Barriers to the Wider Use of Pre-exposure Prophylaxis in the United States: A Narrative Review. Adv Ther. 2020;37(5):1778-811.

22. Lankowski AJ, Bien-Gund CH, Patel VV, Felsen UR, Silvera R, Blackstock OJ. PrEP in the Real World: Predictors of 6-Month Retention in a Diverse Urban Cohort. AIDS Behav. 2019;23(7):1797-802.

23. Clifton S, Nardone A, Field N, Mercer CH, Tanton C, Macdowall W, et al. HIV testing, risk perception, and behaviour in the British population. Aids. 2016;30(6):943-52.

24. Coyer L, van den Elshout MAM, Achterbergh RCA, Matser A, Schim van der Loeff MF, Davidovich U, et al. Understanding pre-exposure prophylaxis (PrEP) regimen use: Switching and discontinuing daily and event-driven PrEP among men who have sex with men. EClinicalMedicine. 2020;29-30:100650.

25. Pasipanodya EC, Jain S, Sun X, Blumenthal J, Ellorin E, Corado K, et al. Trajectories and Predictors of Longitudinal Preexposure Prophylaxis Adherence Among Men Who Have Sex With Men. J Infect Dis. 2018;218(10):1551-9.

26. Wood S, Gross R, Shea JA, Bauermeister JA, Franklin J, Petsis D, et al. Barriers and Facilitators of PrEP Adherence for Young Men and Transgender Women of Color. AIDS Behav. 2019;23(10):2719-29.

27. Yun K, Xu JJ, Zhang J, Li JM, Hu QH, Chu ZX, et al. Female and younger subjects have lower adherence in PrEP trials: a meta-analysis with implications for the uptake of PrEP service to prevent HIV. Sex Transm Infect. 2018;94(3):163-8.

28. Morgan E, Ryan DT, Newcomb ME, Mustanski B. High Rate of Discontinuation May Diminish PrEP Coverage Among Young Men Who Have Sex with Men. AIDS Behav. 2018;22(11):3645-8.

29. Reyniers T, Hoornenborg E, Vuylsteke B, Wouters K, Laga M. Pre-exposure prophylaxis (PrEP) for men who have sex with men in Europe: review of evidence for a much needed prevention tool. Sexually Transmitted Infections. 2017;93(5):363-7.

30. Zablotska I, Grulich AE, Phanuphak N, Anand T, Janyam S, Poonkasetwattana M, et al. PrEP implementation in the Asia-Pacific region: opportunities, implementation and barriers. Journal of the International AIDS Society. 2016;19(7S6):21119.

31. Irungu EM, Baeten JM. PrEP rollout in Africa: status and opportunity. Nature Medicine. 2020;26(5):655-64.

32. Laurent C, Dembélé Keita B, Yaya I, Le Guicher G, Sagaon-Teyssier L, Agboyibor MK, et al. HIV pre-exposure prophylaxis for men who have sex with men in west Africa: a multicountry demonstration study. Lancet HIV. 2021;8(7):e420-e8.

33. Wahome E, Boyd A, Thiong'o AN, Mohamed K, Oduor T, Gichuru E, et al. Stopping and restarting PrEP and loss to follow-up among PrEP-taking men who have sex with men and transgender women at risk of HIV-1 participating in a prospective cohort study in Kenya. HIV Med. 2022;23(7):750-63.

34. Jourdain H, de Gage SB, Desplas D, Dray-Spira R. Real-world effectiveness of pre-exposure prophylaxis in men at high risk of HIV infection in France: a nested case-control study. The Lancet Public Health. 2022;7(6):e529-e36.

35. Scott HM, Spinelli M, Vittinghoff E, Morehead-Gee A, Hirozawa A, James C, et al. Racial/ethnic and HIV risk category disparities in preexposure prophylaxis discontinuation among patients in publicly funded primary care clinics. AIDS. 2019;33(14):2189-95.

36. Thornton A, Lee P. Publication bias in meta-analysis: its causes and consequences. Journal of Clinical Epidemiology. 2000;53(2):207-16.

# Supporting information

**S1 Table 1. Search strategy.**

**S2 Table. Risk of bias assessment using the Newcastle-Ottawa Scale (NOS) for assessing the quality of nonrandomized studies in this systematic review and meta-analyses (this table also includes the citations for every study; see supplementary reference list 1 for references).** *Since we analyzed RCT studies as cohort studies, these too have been evaluated with the Newcastle-Ottawa Quality Assessment Scale for cohort studies rather than with an RCT specific tool.

**S3 Table. Characteristics of included studies.** Abbreviations: MSM=men who have sex with men; TGP=transgender person; PrEP=pre-exposure prophylaxis; IQR=interquartile range; N.A.=not applicable because missing or not reported.

**S4 Table. Description of study characteristics according to region of study.** Data retrieved from 90 studies conducted between 2010 and 2020; three studies conducted in multiple geographic regions were not included in this analysis. All continuous variables are summarized as median [IQR]. Abbreviations: IQR=interquartile range; MSM=men who have sex with men; TGP=transgender person; PrEP=pre-exposure prophylaxis; STI=sexually transmitted infection. *Defined as the year in which the study with PrEP follow-up was initiated; data missing for 8 studies: North America (n=4), Australia (n=1), South America (n=1), Africa (n=2). †Defined as the year of last reported follow-up in the study; data missing for 8 studies:

North America (n=4), Australia (n=1), South America (n=1), Africa (n=2). [‡]Missing data or nonapplicable study population in 8 studies for MSM [North America (n=6), South America (n=2)], 45 for TGP [North America (n=30), Europe (n=1), Australia (n=3), South America (n=3), Africa (n=4), Asia (n=4)] and 63 for other populations [North America (n=35), Europe (n=4), Australia (n=3), South America (n=6), Africa (n=5), Asia (n=10)]. [ǁ]Missing data in 54 studies: North America (n=37), Australia (n=2), South America (n=5), Africa (n=4), Asia (n=6). [¶]Defined as the terminology used in the study to indicate or determine PrEP retention. [$]Only one study with reported data.

**S5 Table. Table of primary retention outcomes.** Abbreviations: N.A.=not applicable in case of missing/non-reported data.

**S1 Fig. Funnel plots assessing publication bias in studies on discontinuing pre-exposure prophylaxis for preventing HIV.** Observed outcome (i.e., ln(-ln) transformed probability of remaining on PrEP) and standard errors corresponding to ln(-ln) transformed probabilities are plotted at month 3 (A), month 6 (B), month 9 (C), and month 12 (D) after PrEP initiation. Asymmetry in the plots was tested using Egger's test for bias. At each timepoint, it was apparent that larger studies (i.e., those with lower standard errors) exhibited higher cumulative probabilities of remaining on PrEP. This bias was significant at each timepoints (month 3, p<0.0001; month 6, p<0.0001; month 9, p=0.0005; month 12, p<0.0001)**.**

**S1 References. References of included papers and abstracts (cited in S2 Table).**

**S1 PRISMA checklist. PRISMA checklist**

**Fig 1. Flow diagram of study search and inclusion.**

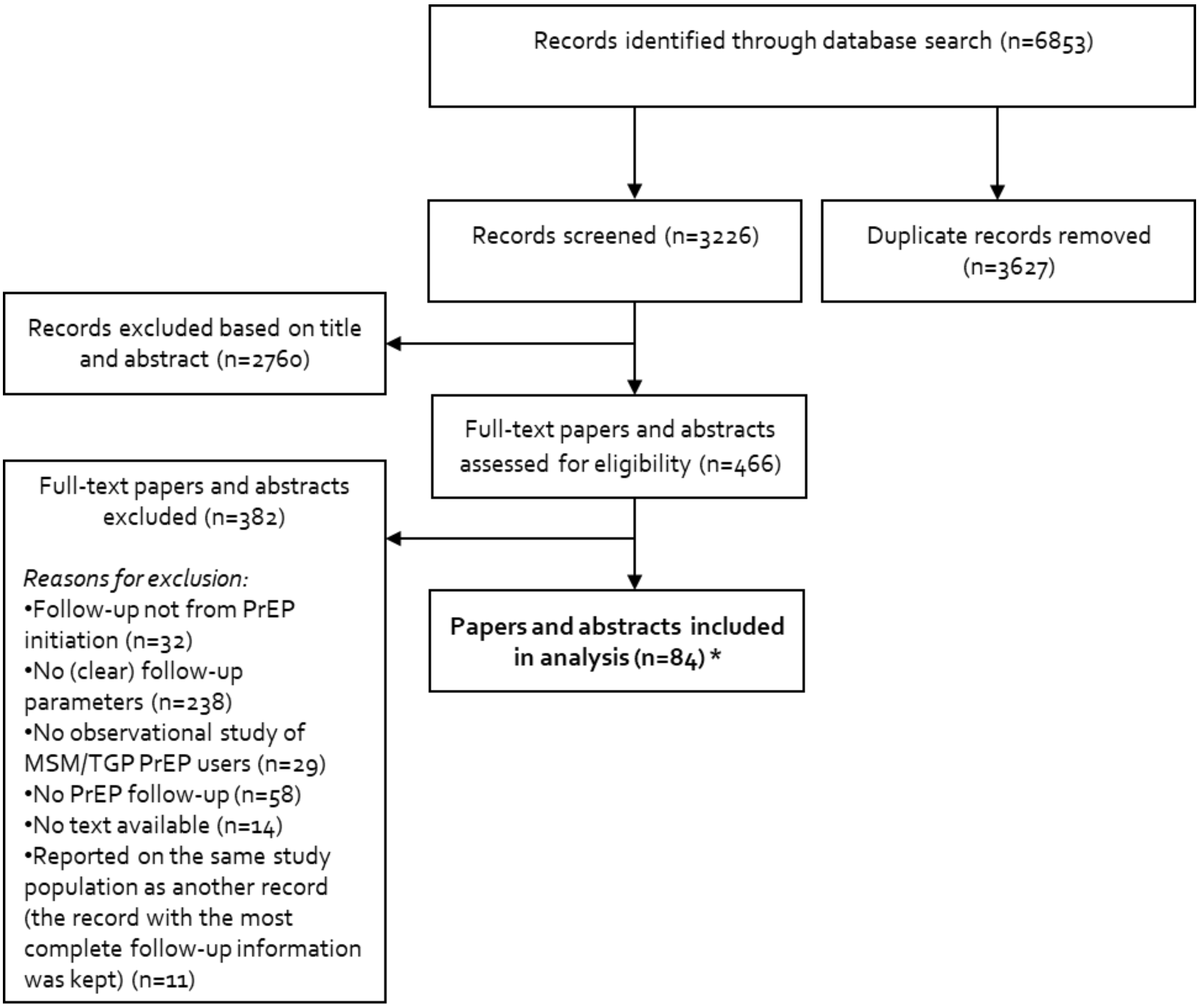


*Studies with two study arms (n=6) were included as one study but were analyzed separately; in total, the number of inclusions is therefore 84 individual studies but this amounts to 90 inclusions (i.e., study cohorts) in the analyses. Abbreviations: MSM=men who have sex with men; TGP=transgender person; PrEP=pre-exposure prophylaxis.

Click here to access/download;Figure;Fig2.docx

**Fig 2. Cumulative probability of individuals remaining on pre-exposure prophylaxis for preventing HIV, over time.**

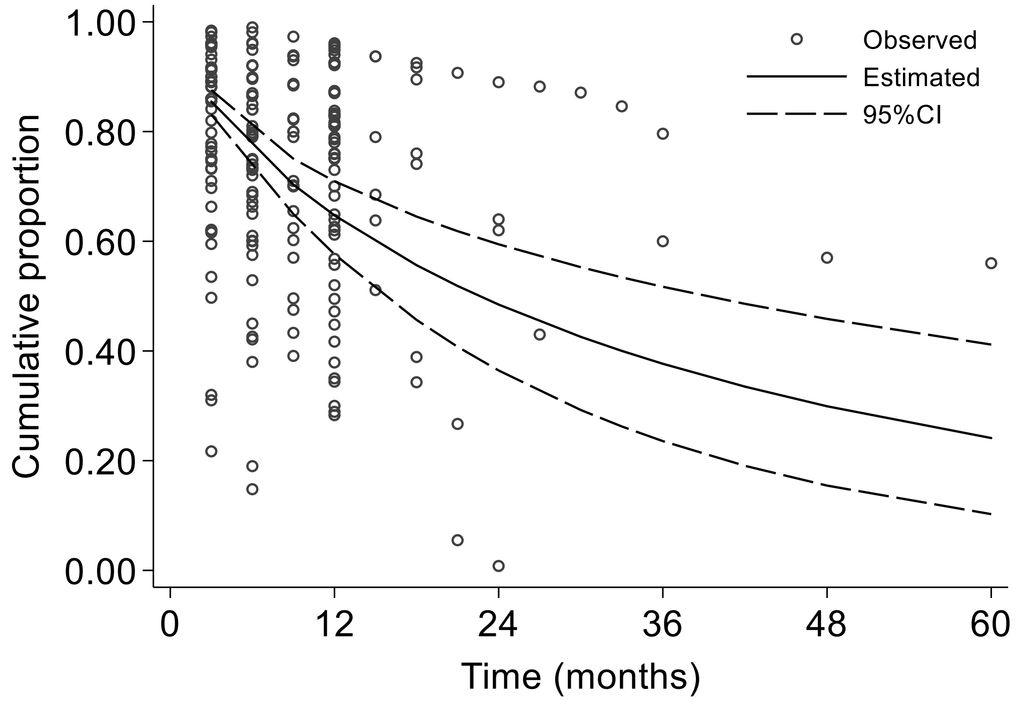


Data retrieved from 90 studies conducted between 2010 and 2020 and ranging from 3 to 60 months of follow-up (median=12). Individual values for studies are depicted as grey dots.

Click here to access/download
**Supporting Information**
S1_PRISMA checklist.pdf

Click here to access/download
**Supporting Information**
S1_References.docx

Click here to access/download
**Supporting Information**
S1_Fig.docx

Click here to access/download
**Supporting Information**
S5_Table.docx

Click here to access/download
**Supporting Information**
S4_Table.docx

Click here to access/download
**Supporting Information**
S3_Table.docx

Click here to access/download
**Supporting Information**
S2_Table.docx

Click here to access/download
**Supporting Information**
S1_Table.docx
