# Supplementary material for: Oral pre-exposure prophylaxis retention among men who have sex with men and transgender persons: Systematic review and meta-analysis
Source: PLoS One. 2025 Oct 17;20(10):e0333494. doi: 10.1371/journal.pone.0333494 (PMC12533894; doi:10.1371/journal.pone.0333494)
Supplement: Attachment — Submitted filename: Response to Reviewers.docx [file pone.0333494.s011.docx]

**From:** Feline de la Court, MSc

Department of Infectious Diseases, Research and Prevention

Public Health Service of Amsterdam

Nieuwe Achtergracht 100, 1018 WT Amsterdam, the Netherlands

**To:** Septime P.H. Hessou, M.D.

Academic Editor

PLOS ONE

Re: ***Revision of manuscript and author response to reviewer comments***

Dear Dr. Septime P.H. Hessou,

We thank you for considering our manuscript, entitled “Long-term pre-exposure prophylaxis retention among men who have sex with men and transgender persons: systematic review and meta-analysis”, and we wish to thank the reviewers for their valuable comments. Our appreciation goes out to their time and effort. We have carefully considered each comment and have edited the manuscript accordingly. The reviewer comments were insightful and have helped improve the clarity and completeness of our manuscript.

Please find our responses to each of the reviewers’ comments below. Revisions in the revised manuscript and author responses are indicated in track changes, referenced by line numbers (found in the revised manuscript with tracked changes). An unmarked version of the revised paper has also been provided.

The Acknowledgements section, Competing Interests section and Data Availability statement have each been updated as specified in the responses in the section Journal Requirements found below.

We would like update our Funding Statement to read as follows:

“The current study is part of the OptiPrEP (optimizing pre-exposure prophylaxis roll-out among men having sex with men) project, funded by the Aidsfonds (P-54601).

“The funders had no role in study design, data collection and analysis, decision to publish, or preparation of the manuscript.”

We would also like to update our Competing interests to read as follows:

“MP obtained unrestricted research grants and speaker/ advisory fees from Gilead Sciences, Abbvie and MSD; all of which were paid to her institute and were unrelated to the current work. EH obtained unrestricted research grants from Gilead Sciences, which were paid to her institute and were unrelated to the current work. The institution of MFSvdL receives study funding from GSK; he served on advisory boards of GSK and Merck/MSD; fees were paid to his institution. AB has received speaker fees from Gilead Sciences, which was unrelated to the current work. This does not alter our adherence to PLOS ONE policies on sharing data and materials. The other authors report no conflicts of interest.

Finally, David O.T. ten Hoff, whose contribution to the manuscript had been formerly mentioned in the acknowledgements, was able to fulfill the ICJME criteria for authorship. He was unfortunately severely ill during the submission process and was unable to submit any confirmation of authorship to *PLOS ONE*. It is with great sadness to report that he passed away when the manuscript was being reviewed. We would then like to add him posthumously as a co-author.

We hope that the revisions in our manuscript and accompanying responses to each comment have shaped our manuscript in such a way that it is suitable for publication in *PLOS ONE*. We greatly look forward to your response.

Sincerely,

On behalf of the authors,

Feline de la Court

E-mail: fdlcourt@ggd.amsterdam.nl

telephone: +31 (0)6 37 42 35 06

**JOURNAL REQUIREMENTS**

1. Please ensure that your manuscript meets PLOS ONE's style requirements, including those for file naming.

**Re: Our manuscript has been checked and meets PLOS ONE’s style requirements, including those for file naming.**

2. We note that you have provided additional information within the Acknowledgements Section that is not currently declared in your Funding Statement. Please note that funding information should not appear in the Acknowledgments section or other areas of your manuscript. We will only publish funding information present in the Funding Statement section of the online submission form.

Please remove any funding-related text from the manuscript and let us know how you would like to update your Funding Statement.

Please include your amended statements within your cover letter; we will change the online submission form on your behalf.

**Re: Apologies, all funding information has been removed from the manuscript and all modifications of the Funding statement have been included in the cover letter.**

3. The authors have declared that no competing interests exist.

Please confirm that this does not alter your adherence to all PLOS ONE policies on sharing data and materials, by including the following statement: "This does not alter our adherence to PLOS ONE policies on sharing data and materials.” (As detailed online in our guide for authors http://journals.plos.org/plosone/s/competing-interests). If there are restrictions on sharing of data and/or materials, please state these. Please note that we cannot proceed with consideration of your article until this information has been declared.

Please include your updated Competing Interests statement in your cover letter; we will change the online submission form on your behalf.

**We confirm that this does not alter our adherence to all PLOS ONE policies on sharing data and materials. Modifications of the Conflict-of-interest section have been included in the cover letter and changed in the manuscript (lines 549-558):**

| **Original text** | **Revised text** |
| --- | --- |
| MP obtained unrestricted research grants and speaker/ advisory fees from Gilead Sciences, Abbvie and MSD; all of which were paid to her institute and were unrelated to the current work. EH obtained unrestricted research grants from Gilead Sciences, which were paid to her institute and were unrelated to the current work. The institution of M. F. Schim van der Loeff receives study funding from GSK; he served on advisory boards of GSK and Merck/MSD; fees were paid to his institution. The other authors report no conflicts of interest. | MP obtained unrestricted research grants and speaker/ advisory fees from Gilead Sciences, Abbvie and MSD; all of which were paid to her institute and were unrelated to the current work. EH obtained unrestricted research grants from Gilead Sciences, which were paid to her institute and were unrelated to the current work. The institution of M. F. Schim van der Loeff receives study funding from GSK; he served on advisory boards of GSK and Merck/MSD; fees were paid to his institution. **AB has received speaker fees from Gilead Sciences, which was unrelated to the current work. This does not alter our adherence to PLOS ONE policies on sharing data and materials.** The other authors report no conflicts of interest. |

4. In your Data Availability statement, you have not specified where the minimal data set underlying the results described in your manuscript can be found. PLOS defines a study's minimal data set as the underlying data used to reach the conclusions drawn in the manuscript and any additional data required to replicate the reported study findings in their entirety. All PLOS journals require that the minimal data set be made fully available. For more information about our data policy, please see http://journals.plos.org/plosone/s/data-availability.

Upon re-submitting your revised manuscript, please upload your study’s minimal underlying data set as either Supporting Information files or to a stable, public repository and include the relevant URLs, DOIs, or accession numbers within your revised cover letter. For a list of acceptable repositories, please see http://journals.plos.org/plosone/s/data-availability#loc-recommended-repositories. Any potentially identifying patient information must be fully anonymized.

We will update your Data Availability statement to reflect the information you provide in your cover letter.

**Re: We have made the following changes to the Data availability statement:**

| **Original text** | **Revised text** |
| --- | --- |
| All data and analyses used in this study are available on GitHub. | **The minimal dataset underlying the results and the statistical analysis code used in this study are available on GitHub, <https://github.com/boyd0094/GGD_PrEP_retention>** |

**Please note that this link will not work until formal acceptance of publication.**

5. Please review your reference list to ensure that it is complete and correct.

**Re: Our reference list has been checked and it is both complete and correct.**

**EDITOR REMARKS**

1. It would be wise to clearly define in the methodology what long-term retention means in terms of procedure, type of PrEP and timeframe. Especially as PrEP must be continuous at all times to avoid any risk.

**Re: We thank the editor for this helpful comment. In the manuscript, we used the term “long-term” in several contexts, which was rather ambiguous. Considering that retention was defined in various ways across studies, not all of them describing long-term retention, we decided to remove this adjective altogether when describing retention in both the title and text.**

**One additional comment: to help avoid the risk of HIV, engagement in PrEP care needs to be continuous, not PrEP itself. PrEP can be taken around the time of at-risk exposures and still be fully effective in preventing HIV (see Molina J-M, et al, *NEJM*, 2015 or Hoornenborg E et al, *Lancet HIV*, 2020).**

2. Introduction: At this level, it would be good to already hypothesize about program characteristics that may influence target retention on PrEP. The absence of this made it difficult to understand your results and conclusions, as this was the aim of the study.

**Re: Although we generally agree that hypotheses should be stated clearly prior to conducting any study, the fact that this meta-analysis includes a large number of determinants makes it difficult not only to introduce a hypothesis for each individual determinant, but also the literature that would lead up to these hypotheses.**

**For the sake of brevity, we summarized the factors that could influence PrEP retention in the introduction.**

3. Methods: In this methodological part, as you have used PRISMA, please present clearly: PICOS

P= Populations, I= Interventions, C=comparator, O= outcome, S= Study

**Re: We have now clearly mentioned each of these aspects in the section on inclusion criteria (lines 108-126):**

| **Original text** | **Revised text** |
| --- | --- |
| We included only studies that reported on retention of people using oral PrEP for HIV (i.e., fixed dose combination of emtricitabine and tenofovir disoproxil fumarate) taken daily, event-driven or both, and that were conducted among a predominantly (≥60%) MSM and/or TGP population. Moreover, studies were included if follow-up time and proportion of participants retained in care from PrEP initiation to discontinuation were reported or could be calculated for at least one of the pre-defined follow-up time points (i.e., beginning at month 3 since PrEP initiation, and continuing for every 3 months until month 60). Eligible study designs were: unblinded randomized controlled trials (RCT), open label extension (OLE) studies, longitudinal observational cohort studies, demonstration projects, implementation projects, and studies conducted in primary care or other routine public health settings. | We included studies **conducted in predominantly MSM and/or TGP populations (i.e., ≥60% of the study population consisted of these key populations) who were using oral PrEP for HIV** (i.e., fixed dose combination of emtricitabine and tenofovir disoproxil fumarate) taken daily, event-driven or both. **No comparison between regimens was required for inclusion.** **Studies were required to provide a quantitative measure of retention on PrEP.** **We categorized definitions of retention related to the following: PrEP continuation, where PrEP was still being used by the participant according to self-reports, prescription data, etc.; follow-up, where participants were still actively in care or enrolled in a study; mixed/other, where composite definitions or a definition other than continuation or follow-up were used.** Studies were **therefore** included if follow-up time and proportion of participants **retained from PrEP initiation to end of follow-up** were reported or could be calculated for at least one of the pre-defined follow-up time points (i.e., beginning at month 3 since PrEP initiation, and continuing for every 3 months until month 60). Eligible study designs were: unblinded randomized controlled trials (RCT), open label extension (OLE) studies, longitudinal observational cohort studies, demonstration projects, implementation projects, and studies conducted in primary care or other routine public health settings. |

4. Data Extraction: How is it possible to do a follow-up or retention study with on-demand or event-related PrEP?

**Re: Good point. Regardless of PrEP modality (continuous or event-related), individuals on PrEP need to be regularly screened for STIs and ensure that they did not acquire HIV. PrEP retention here would be defined as an individual who continues regularly screening visits.**

**We need to point out that different definitions of retention were used across studies. (This issue is why we decided to include it as a covariate in the meta-analysis.) Some studies did use continuing PrEP pills as a definition of retention, which would only be applicable to continuous PrEP.**

**It should be noted that we had separate definitions labelled “retention” and “follow-up” in our previous submission. These are nearly identical and given the broad use of retention in the manuscript, we have labeled this as “follow-up”.**

**We have made it clearer why definitions are important in the introduction (lines 70-76):**

| **Original text** | **Revised text** |
| --- | --- |
| Retention outcomes may also be attributable to factors related to location, financing and implementation or research in PrEP care[9-11]. So far, no previous review study has provided an aggregated analysis on the extent to which such program-specific characteristics affect PrEP retention outcomes. | Retention outcomes may also be attributable to factors related to location, financing and implementation or research in PrEP care[9-11]. **Retention outcomes could also vary with the type of oral PrEP being used (i.e., daily or event/driven), while certain definitions of retention, such as PrEP intake or PrEP prescription, no longer apply to those using event-driven PrEP.** So far, no previous review study has provided an aggregated analysis of the extent to which such program-specific characteristics, **including specific definitions of retention**, affect PrEP retention outcomes. |

**We have also provided more information on what the terms for discontinuation terms represented (lines 115-119):**

| **Original text** | **Revised text** |
| --- | --- |
| **--** | **We categorized definitions of retention related to the following: PrEP continuation, where PrEP was still being used by the participant according to self-reports, prescription data, etc.; follow-up, where participants were still actively in care or enrolled in a study; mixed/other, where composite definitions or a definition other than continuation or follow-up were used.** |

5. Results: For RCTs, how was the analysis done if the arm does not contain the drug, is it ethical please?

**Re: RCT arms without PrEP were not included in the analysis. This applied to very few studies in our analysis. We made this clearer in the methods section (lines 132-134):**

| **Original text** | **Revised text** |
| --- | --- |
| For open-label RCTs containing both an “intervention” and “standard of care” arm, each arm was included as a separate study cohort. | For open-label RCTs containing both an “intervention” and “standard of care” arm, each arm was included as a separate study cohort **if the arm included individuals using PrEP**. **If the arm did not include individuals using PrEP, it was not included in the study.** |

**These RCTs were conducted at a time when the efficacy of PrEP was completely unknown, hence they were ethical at the time of completion.**

**Of note, the included RCTs did not pertain to groups using versus not using PrEP. The RCTs commonly compared other interventions, such as apps/consulting etc., in addition to PrEP care versus PrEP care alone (stated as “standard of care” in the table summarizing studies).**

6. The problem posed by the discussion is that of the completeness of the studies and consequently of the extrapolation of the results: the majority of the studies are from North America, just 5% for each of the other parts of the world, so the discussion of this representativeness bias needs to be strengthened.

**Re: We fully agree. The following sentence has been added to the discussion (lines 498-501):**

| **Original text** | **Revised text** |
| --- | --- |
| **--** | **“Fifth, the vast majority of published studies represented European, Australian and North American settings. Although some studies from South East Asia, Africa and South America were included, the pooled estimates might not be fully representative of these and other non-included regions.”** |

7. Reinforce the conclusion with program factors that actually influence MSM and TG retention on PrEP.

**Re: We have added a list of program-related factors that could help reinforce PrEP retention in the conclusion (lines 527-530):**

| **Original text** | **Revised text** |
| --- | --- |
| **--** | **PrEP programs with more intense monitoring schedules or requiring that patients pay for some of their PrEP/STI testing services may face lower rates of retention; however, clearer evidence that these program factors do indeed have an effect on retention is needed.** |

**REVIEWER 1**

1. It includes data on event driven PrEP but it is unclear how many participants were receiving event driven PrEP. For about 40% this is unspecified. Retention in event driven PrEP is a slight misnomer. And the paper does not include different definitions of retention for event driven and daily PrEP which I think is warranted.

**Re: This is a very good point. The studies included used various definitions of “retention.” We were able to categorize retention using three different types of definitions: continuation, where PrEP was still being used by the participant according to self-reports, prescription data, etc.; follow-up, where participants were still actively in care or enrolled in a study; mixed/other, where composite definitions or a definition other than continuation or follow-up were used. Given the differences in definitions, we felt it important to include as a covariate in our meta-regression.**

**We have decided to add clarification in the methods that studies with any quantitative measure of PrEP retention were included (lines 115-119):**

| **Original text** | **Revised text** |
| --- | --- |
| **--** | **We categorized definitions of retention related to the following: PrEP continuation, where PrEP was still being used by the participant according to self-reports, prescription data, etc.; follow-up, where participants were still actively in care or enrolled in a study; mixed/other, where composite definitions or a definition other than continuation or follow-up were used.** |

**Most studies seemed to choose definitions related to follow-up or mixed/other (n=72/88, 81.8%), which could easily apply to both PrEP modalities. Nevertheless, some studies (n=7/35, 20.0%) including unspecified PrEP modality used PrEP continuation as a measure of retention. This is unlikely to represent true retention of event-driven PrEP users, if they were included. Even more problematic is that very few of the studies with combined PrEP modalities stratified their PrEP retention measures on daily or event-driven PrEP, making it difficult to include in our analysis.**

**We have provided some results on the frequencies of definitions within certain PrEP modalities (lines 281-284):**

| **Original text** | **Revised text** |
| --- | --- |
| **--** | **Definitions of retention were predominately based on follow-up (n=56; 62.9%). Definitions related to continuation were used less frequently (n=16; 18.0%), and found in n=7/47 (14.9%) studies with daily PrEP, n=0/1 (0%) with event-driven PrEP, n=2/5 (40.0%) with mixed daily and event-driven PrEP, and n=7/35 (20.0%) with unspecified PrEP modality.** |

**We have also reshaped some of the discussion about the problems with definitions vis-à-vis PrEP modality in the Discussion (lines 389-408):**

| **Original text** | **Revised text** |
| --- | --- |
| It should be noted that PrEP discontinuation is not per se an undesirable endpoint. If individuals have a low likelihood or low perceived likelihood of acquiring HIV, discontinuation is warranted with appropriate counselling[18]. Research shows that those who discontinue PrEP when chances of acquiring HIV are low, may be more inclined to re-initiate when the likelihood of HIV increases again[19, 20]. Hence, efforts to improve PrEP retention should focus on the groups discontinuing PrEP while still substantially vulnerable for HIV. That being said, re-initiation with increased HIV likelihood implies timely access to PrEP, which is not always the case, and counselling after discontinuation may also lead to HIV prevention modes other than PrEP; highlighting the importance of tailored and well-timed PrEP counselling. It is also important to consider that the definitions used in studies may also shape the way discontinuation is addressed. Our findings show that studies who use the term ‘retention’ rather than ‘discontinuation’ had higher rates of PrEP discontinuation (albeit non-significantly). Further research comparing the ways in which programs encourage retention, or conversely, avoid discontinuation, and how definitions shape outcomes could clarify this finding. | **It is important to consider that retention is defined in different ways across studies, but differences in definitions seem to have little effect on loss of retention rates. For daily PrEP, continuation of PrEP intake could be an appropriate measure of retention. However,** PrEP discontinuation is not per se an undesirable endpoint. If individuals have a low likelihood or low perceived likelihood of acquiring HIV, discontinuation is warranted with appropriate counselling[18]. Research shows that those who discontinue PrEP when chances of acquiring HIV are low, may be more inclined to re-initiate when the likelihood of HIV increases again[19, 20]. **This type of dosing would be essentially event-driven PrEP, which would require other measures of retention, such as those based on follow-up. Retention in PrEP care could be particularly helpful as** timely access to PrEP and counselling after discontinuation **are essential to ensure HIV prevention**. |

2. Suggest including details about daily vs event driven PrEP in the intro

**Re: Thank you for the suggestion. We have added brief mention of the two PrEP modalities in the Introduction (lines 50-51):**

| **Original text** | **Revised text** |
| --- | --- |
| **--** | **PrEP can be taken either every 24 hours (i.e., daily) or before and after sexual contact (i.e., event-driven).** |

**We have also proposed that the type of PrEP modality could have an effect on retention in the Introduction (lines 71-74):**

| **Original text** | **Revised text** |
| --- | --- |
| **--** | **Retention outcomes could also vary with the type of oral PrEP being used (i.e., daily or event/driven), while certain definitions of retention, such as PrEP intake or PrEP prescription, no longer apply to those using event-driven PrEP.** |

3. Is there information missing from table 1? I cannot tell how many studies in each of the calendar year brackets.

**Re: This information was explained in the footnote. For clarity, we have included the number of studies to which these statistics apply along with the category titles in Table 1:**

| **Original text** | **Revised text** |
| --- | --- |
| Year begun*  Year completed†  Year published | Year begun **[N=82]***  Year completed **[N=82]**†  Year published **[N=90]** |

4. In table 2 - I am unclear how to interpret the rows on percentage of study population (%). Can this be better displayed and explained in the text?

**Re: Our apologies. Explanation of these percentages have been added to the footnote in Table 1:**

| **Original text** | **Revised text** |
| --- | --- |
| -- | **‡The percentage of study population is defined at the total number of individuals in a given key population (e.g., MSM, TGP) divided by the total number of individuals included in the study. Some studies also calculated this percent in other groups, which are included in this table.** |

5. Sometimes the sentence structure is confusing to me. For example, "PrEP discontinuation was significantly

lower as median age of participants was lower (p=0.035)." Clearer to say: Younger median age of participants was associated with continuing on PrEP. Although this seems at odds with this statement in the discussion: "We found that with older age, the rates of PrEP discontinuation were lower." I think the whole paper could do with an edit.

**Re: Thank you for bringing up this issue. Some of the confusion might come from the word order. We have now changed it in the text by stating the determinant first and outcome second. There was also an error in the referenced sentence and it should have been “higher” age, rather than “lower” age. The following changes were made in the Results (lines 337-339):**

| **Original text** | **Revised text** |
| --- | --- |
| PrEP discontinuation was significantly  lower as median age of participants was lower (p=0.035). | **Higher median age of participants was associated with decreased loss of retention rates** (*p*=0.035). |

**The rest of the paragraph has also been edited, accordingly. We have now used more consistent terminology for clarity.**

6. Unclear why the definition of outcome was included as a variable. I cannot understand why definition would impact outcome and it is not well explained. I suggest excluding this from the paper.

**Re: We kindly request the reviewer to refer to their first comment, which explains the rationale for including retention definition in the analysis.**

**REVIEWER 2**

1. The search has not been updated since end of March 2021. Many more studied could have published since that date that could be included here.

**Re: We appreciate the reviewer’s comment. We do recognize that given the date of article retrieval, we could have missed some studies. Nevertheless, including articles after this date would likely include studies during the COVID-19 pandemic, where PrEP allocation and PrEP taking patterns could be different and thus leading to increased heterogeneity in PrEP retention rates. We admit that there could be other manuscripts on PrEP retention from the period prior to the COVID-19 pandemic published after March 2021. However, since we additionally searched conference abstracts, where data on PrEP retention are highly likely to be presented, the risk of not including these other studies should be reduced.**

**For these reasons, we have decided not to update this systematic review using a more recent date.**

2. Aa a corollary to point #1, authors could have investigated whether retention rates have changed in recent years after the introduction of long-lasting regimens for PrEP.

**Re: Excellent point. Approval for the use of long-acting injectable PrEP (i.e., cabotegravir) is very recent. Only the US approved this drug in 2021, with Australia following in 2022 and the European Union in 2023 (data from <https://www.prepwatch.org/>). As a result, real world data are severely lacking and any comparison between the available data from the randomized control trials on cabotegravir PrEP to our data on implementation and pilot projects using oral PrEP might not be valid. Furthermore, cabotegravir PrEP has been recommended as an option for individuals at very high risk of HIV acquisition (from the most recent WHO guidelines: <https://www.who.int/publications/i/item/9789240054097>) and many HIV prevention programs are struggling with implementing cabotegravir PrEP (see Liegeon & Ghosn, HIV Med. 2023 Jun;24(6):653-663). This topic would be more appropriately addressed when more data become available.**

**Nevertheless, we have now stated throughout the manuscript that we evaluated oral PrEP. We have also added a sentence in the conclusion stating that further research on cabotegravir PrEP is needed (lines 503-509):**

| **Original text** | **Revised text** |
| --- | --- |
| **--** | **We only included studies using oral PrEP, meanwhile injectable cabotegravir can also be used to effectively prevent HIV acquisition. At the end of 2023, cabotegravir PrEP has been recommended as an option for individuals at very high risk of HIV acquisition [38], but many HIV prevention programs seem to be struggling with implementing cabotegravir PrEP [39]. Further research on PrEP retention would be needed for the use of cabotegravir PrEP, particularly when more observational data become available.** |

3. According to the 'star system' for the Newcastle-Ottawa Quality Assessment Scale studies, among other things, are judged on the comparability of the groups. The meaning of using this system to judge RCTs in unclear as by definition groups are comparable because of randomization.

**Re: Excellent point. Admittedly, this item was difficult to evaluate for randomized control trials.**

**This criterion reads, “Comparability of cohorts on the basis of the design or analysis.” Randomization is meant to control for comparability between arms, hence it would correspond with the design aspects of the study. However, our research question was specific to only rates of PrEP retention regardless of exposure (i.e., single arm studies) and we were more interested in the analysis aspects of studies.**

**As a result, we evaluated this criterion as whether these rates were adjusted for age and (if diverse populations were included) gender (one star), and whether these rates were additionally adjusted for other factors (one star). We have added the following to the footnote of Supplementary Table S2 to clarify this:**

| **Original text** | **Revised text** |
| --- | --- |
| **--** | **†We evaluated comparability on the basis of the analysis and not the design (as studies were single-arm). This evaluation was carried out as follows: whether proportions were adjusted for age and (if diverse populations were included) gender (one star), and whether proportions were additionally adjusted for other factors (one star). The comparability criteria were applied similarly for randomized control trials and observational studies.** |

4. Because of possible publication bias and potential confounding, I would perform a sensitivity analysis after restricting only to the RCTs. Similarly, I would like to see the results of a sensitivity analysis after excluding studies with small sample size.

**Re: Agreed and thank you kindly for this suggestion. These analyses have been added to the Methods (lines 196-203):**

| **Original text** | **Revised text** |
| --- | --- |
| **--** | **In sensitivity analyses, we generated separate curves while only including randomized control studies (to assess bias from observational data), studies with more than 100 participants (to assess the effect of potential publication bias), and studies with a Newcastle-Ottawa Quality Assessment Scale risk score bias of more than 5. Given that most studies collected data until month 24 of follow-up, we also generated the curve using data up until month 24 to assess any change in model fit. The model was slightly modified for some of these sensitivity analyses for purposes of convergence: variance-covariance structure was exchangeable when including only randomized-control studies.** |

**These results have also been described in the Results (lines 293-306):**

| **Original text** | **Revised text** |
| --- | --- |
| **--** | **Sensitivity analysis demonstrated that the pooled cumulative probability of PrEP retention was higher when including only randomized control trials (Fig 2B), relatively unchanged for only studies with more than 100 participants (Fig 2C), and mostly unchanged for only studies with a risk score bias of less than 5 (Fig 2D). For randomized control trials only, the pooled cumulative probability of PrEP retention was 77.3% (95%CI=68.9-83.6%) at 6 months, 71.1% (95%CI=56.7-81.5%) at 12 months, and 63.8% (95%CI=40.1-80.2%) at 24 months. There were no other studies beyond this time point. For studies with more than 100 participants only, the pooled cumulative probability of PrEP retention was 79.0% (95%CI=75.1-82.4%) at 6 months, 67.6% (95%CI=60.0-74.1%) at 12 months, 52.1% (95%CI=38.9-63.8%) at 24 months, and 27.9% (95%CI=11.6-47.0%) at 60 months.** |

**Some issues to mention:**

- **There were very few pure RCTs. There were a handful of RCTs that then turned into open-label studies. Given the few numbers of studies, we combined these studies into one category and analyzed them as RCTs.**
- **Defining studies with small sample sizes can be a bit arbitrary. We decided to exclude studies with sample sizes in the lower 25%tile, which was around 100 participants. There is really no statistical backing for this reason, except that these studies seemed to be contributing a fair amount of bias from the funnel plots. Suggestions for other cutoffs are welcome.**

5. Only few studies reported the proportion of retained care after month 24. I suggest that predictions should be given up to month 24 because after that these are based on little data. This also explains why the proportion of retained in care is largely underestimated by the model compared to observed at month 60

**Re: Agreed. These analyses have been added to the Methods (lines 199-201):**

| **Original text** | **Revised text** |
| --- | --- |
|  | **Given that most studies collected data until month 24 of follow-up, we also generated the curve using data up until month 24 to assess any change in model fit.** |

**These results have also been described in the Results (lines 308-311):**

| **Original text** | **Revised text** |
| --- | --- |
|  | **The model also produced similar results when only restricting data collected in the first 24 months (Fig 2E). Using these data, the pooled cumulative probability of PrEP retention was 76.9% (95%CI=73.2-80.1%) at 6 months, 64.6% (95%CI=57.4-70.9%) at 12 months, 48.4% (95%CI=36.1-59.6%) at 24 months.** |

**Interestingly, the estimates remained fairly unchanged, suggesting that the studies after month 24 do not seem to have that much impact on model fit. For this reason, we have decided to keep the analysis of all data points as the main analysis.**

6. Table S2 shows that there were studies with risk score bias of 5. I would like to see the results of a sensitivity analysis after excluding these.

**Re: Thanks again for this suggestion. These analyses have been added to the Methods (lines 196-203):**

| **Original text** | **Revised text** |
| --- | --- |
|  | **In sensitivity analyses, we generated separate curves while only including … studies with a Newcastle-Ottawa Quality Assessment Scale risk score bias of more than 5.** |

**These results have also been described in the Results (lines 293-306):**

| **Original text** | **Revised text** |
| --- | --- |
| **--** | **Sensitivity analysis demonstrated that the pooled cumulative probability of PrEP retention […] and mostly unchanged for studies with a risk score bias of less than 5 (Fig 2D). […] For studies with a risk score bias of more than 5 only, the pooled cumulative probability of PrEP retention was 76.3% (95%CI=72.4-79.8%) at 6 months, 62.8% (95%CI=55.1-69.6%) at 12 months, 45.0% (95%CI=32.4-56.8%) at 24 months, and 19.4% (95%CI=7.0-36.5%) at 60 months.** |

7. There is significant heterogeneity between the studies which could have affected the overall estimate of the retention in care. Authors should consider using the sequential and combinatorial algorithms developed by Patsopoulos et al. [Patsopoulos NA, Evangelou E, Ioannidis JPA. Sensitivity of between-study heterogeneity in meta-analysis: proposed metrics and empirical evaluation. Int J Epidemiol 2008;37:1148−57] which evaluate the change in between-study heterogeneity as one or more studies are excluded from the calculations. The proposed algorithms can be routinely applied in meta-analyses as standardized sensitivity analyses for heterogeneity, and they can either generate the number of studies that have to be omitted to decrease I2 below the specified threshold, or estimate the corresponding proportion of studies.

**Re: Thank you for this very helpful comment. These types of statistical analyses are indeed helpful. We decided to examine studies that could be contributing to heterogeneity using a different method, i.e., GOSH (see Olkin I, Dahabreh IJ, Trikalinos TA, GOSH – a graphical display of study heterogeneity. *Res Syn Meth*, 2012). This method generates multiple combinations of studies with varying sizes and re-estimates the summary statistic and metrics of heterogeneity. The results from this analysis also give us a depiction of whether lower heterogeneity could be achieved. We have added this analysis to the list of diagnostic tests. Since this test was not developed in the framework of the random-effects survival model, we applied this test to data at months 3, 6, 9 and 12:**

**The following changes have been made to the statistical analysis section of the Methods (lines 220-224):**

| **Original text** | **Revised text** |
| --- | --- |
| **--** | **We evaluated whether certain combinations of studies were contributing to excessive heterogeneity using the graphical display of study heterogeneity (GOSH) plot [16]. A total of 106 combinations were randomly selected to produce the graph using the gosh function in the “metafor” package.** |

**The results of these tests have also been added to the Results (lines 370-372):**

| **Original text** | **Revised text** |
| --- | --- |
| **--** | **GOSH plots are provided in S1 Fig, demonstrating no clear combinations of studies that contributed to heterogeneity. From these analyses, the lowest attainable I2 was 89.1% at month 3, 92.5% at month 6, 89.4% at month 9, and 97.0% at month 12.** |

8. Despite the statistical significance some of the effects in the meta-regression analysis (e.g., heterosexuals/women/PWID vs. MSM was only 3% difference) are of dubious clinical significance. In contrast, there may be up to a 2.9-fold increase in discontinuation when PrEP was not free but the meta-regression is underpowered to detect this difference. I would better qualify these findings in the Discussion section.

**Re: Thank you for bringing up this point.**

**To be clear, the 3% difference in HR is per one % increase in the percentage of individuals who are other than MSM or TGP. The effect here is arguably strong for a continuous variable.**

**We very much agree that the effect of not having free oral PrEP (or even free STI testing) is particularly strong despite being underpowered. However, given the few numbers of studies included without free PrEP/STI provisions and the fact that the majority of studies did not have this information, we have stressed, but caveated, these findings.**

**If there are other places where the reviewer feels are not sufficiently or insufficiently stressed, please let us know.**

9. Line 252 – looks like there is a typo. PrEP discontinuation was significantly lower as median age of participants was OLDER (not lower) (p=0.035). There is a 4% lower risk of discontinuation per 1 year older. It does make sense that the younger the participants the higher the rates of discontinuation, not vice versa (as discussed)

**Re: Thank you for the correction. Indeed, this sentence was incorrect. The following changes were made in the Results (lines 337-339):**

| **Original text** | **Revised text** |
| --- | --- |
| PrEP discontinuation was significantly  lower as median age of participants was lower (p=0.035). | **Higher median age of participants was associated with decreased loss of retention rates** (*p*=0.035). |

**REVIEWER 3**

1. The authors refer in the abstract that they will estimate the extent to which variability in PrEP retention is attributable to population- and program-specific characteristics. However, variables such as the type of publication are used. Please, provide the rationale for such inclusion. Additionally, I wondered whether in the objective the authors should use “associated” instead of “attributable”.

**Re: We thank the reviewer for bringing up these points. Indeed, type of publication does not fit in with these characteristics and are more study-specific (as they relate to reporting of results). For meta-analyses, the rationale is straightforward: the quality of data presented in an abstract could be less than a peer-reviewed publication. Given the space limits of the abstract, we did not provide any rationale. We agree with the reviewer that “associated” would be more appropriately suited here.**

**The following changes have been made to the abstract (lines 22-26):**

| **Original text** | **Revised text** |
| --- | --- |
| This study aimed to estimate the proportion of long-term retention in PrEP care and extent to which variability in PrEP retention is attributable to population- and program-specific characteristics among men who have sex with men (MSM) and transgender persons (TGP). | This study aimed to estimate the proportion of retention in PrEP care and extent to which variability in PrEP retention is **associated with** population-, program**-, and study-**specific characteristics among men who have sex with men (MSM) and transgender persons (TGP). |

2. In the results section I would suggest presenting the HR instead of just the p-values which are more informative about the association.

**Re: We understand the reviewer’s comment. We tend to follow the recommendation set forth in the guidance from the International Committee of Medical Journal Editors, “Do not repeat all the data in the tables or figures in the text; emphasize or summarize only the most important observations.” ([https://www.icmje.org/recommendations/browse/manuscript-preparation/preparing-for-submission.html#e](https://www.icmje.org/recommendations/browse/manuscript-preparation/preparing-for-submission.html#e)) We believe that adding HRs would make it cumbersome to read this section when HR are already in the table.**

3. Would it be worth discussing if some determinants were more important in earlier or later studies? I would imagine that both the population and program characteristics may have differed according to the year of study beginning and so the determinants may have also differed.

**Re: This is an excellent point. This question is essentially asking whether there is an interaction between calendar time and each of the individual determinants. These analyses have been added to the Methods (lines 210-212):**

| **Original text** | **Revised text** |
| --- | --- |
| **--** | **To determine whether HRs changed over calendar years, we included calendar year of study start and the interaction term between calendar year of study start and factor to each univariable model, separately, and tested the interaction term.** |

**Of note, we chose the calendar year at which the study began, as publication date might not fully represent when the study was conducted.**

**There was no evidence that any of the HRs changed over calendar time. These results have now been described in the Results (lines 342-343):**

| **Original text** | **Revised text** |
| --- | --- |
| **--** | **There was also no evidence of interaction between year of study start and any of the factors.** |

**IN-TEXT COMMENTS**

1. Abstract: You don't think two databases are enough for a systematic review? If I were you, I'd try to check out Cochrane, Web Science and other unpublished articles.

**Re: We believe that the vast majority of manuscripts on PrEP use would be found in either PubMed or Ovid.**

**In our experience, Web of Science generally provides the same results as PubMed, but can yield more abstracts. Given that we specifically searched abstracts from infectious disease and HIV-related conferences, we believed that Web of Science was unlikely to generate any additional manuscripts.**

**Cochrane might have also generated additional manuscripts, yet the time taken to yield the potentially few articles added through this source would have been rather prohibitive. We have nevertheless added this issue as a limitation (lines 492-495):**

| **Original text** | **Revised text** |
| --- | --- |
| Third, we did not search Embase due to access restrictions and did not include conferences such as the HIV Research for Prevention Conference; hence, although there is likely overlap across these databases/conferences, some studies may have been missed. | Third, we did not search **the Cochrane or** Embase **databases** due to access restrictions and did not include conferences such as the HIV Research for Prevention Conference; hence, although there is likely overlap across these databases/conferences, some studies may have been missed. |

2. Abstract: Is it possible to update the literature review on the databases in November 2023, or at most in December 2022?

**Re: We appreciate the reviewer’s comment. We do recognize that given the date of article retrieval, we could have missed some studies. Nevertheless, including articles after this date would likely include studies during the COVID-19 pandemic, where PrEP allocation and PrEP taking patterns could be different and thus leading to increased heterogeneity in PrEP retention rates. We admit that there could be other manuscripts on PrEP retention from the period prior to the COVID-19 pandemic published after March 2021. However, since we additionally searched conference abstracts, where data on PrEP retention are highly likely to be presented, the risk of not including these other studies should be reduced.**

**For these reasons, we have decided not to update this systematic review using a more recent date.**

3. Abstract: What about injectable PREP? Although it's brand new, you'll be able to talk about it in the introduction, as its advent is directly linked to your research theme.

**Re: Approval for the use of long-acting injectable PrEP (i.e., cabotegravir) is very recent. Only the US approved this drug in 2021, with Australia following in 2022 and the European Union in 2023 (data from <https://www.prepwatch.org/>). As a result, real world data are severely lacking and any comparison between the available data from the randomized control trials on cabotegravir PrEP to our data on implementation and pilot projects using oral PrEP might not be valid. Furthermore, cabotegravir PrEP has been recommended as an option for individuals at very high risk of HIV acquisition (from the most recent WHO guidelines: <https://www.who.int/publications/i/item/9789240054097>) and many HIV prevention programs are struggling with implementing cabotegravir PrEP (see Liegeon & Ghosn, HIV Med. 2023 Jun;24(6):653-663). This topic would be more appropriately addressed when more data become available.**

**Nevertheless, we have now stated throughout the manuscript that we evaluated oral PrEP. We have also added a sentence in the conclusion stating that further research on cabotegravir PrEP is needed (lines 503-509):**

| **Original text** | **Revised text** |
| --- | --- |
| **--** | **We only included studies using oral PrEP, meanwhile injectable cabotegravir can also be used to effectively prevent HIV acquisition. At the end of 2023, cabotegravir PrEP has been recommended as an option for individuals at very high risk of HIV acquisition [38], but many HIV prevention programs seem to be struggling with implementing cabotegravir PrEP [39]. Further research on PrEP retention would be needed for the use of cabotegravir PrEP, particularly when more observational data become available.** |

4. Introduction: At this level, it would be good to already hypothesize about program characteristics that may influence target retention on PrEP. The absence of this made it difficult to understand your results and conclusions, as this was the aim of the study.

**Re: Although we generally agree that hypotheses should be stated clearly prior to conducting any study, the fact that this meta-analysis includes a large number of determinants makes it difficult to introduce not only an hypothesis for each individual determinant, but the literature that would lead up to these hypotheses.**

**For the sake of brevity, we had summarized the factors that could influence PrEP retention in the introduction.**

5. Methods (study design): Very well, but you didn't look for studies in Cochrane, which is the database par excellence for systematic reviews, and also in Web science and other unpublished articles.

**Re: Please refer to comment 1 of the in-text comments.**

6. Methods (search strategy and inclusion): And in the event of a discrepancy between the two [independent reviewers], what has really been done to separate them????

**Re: Our apologies for the lack of clarity. We have provided more information as to what happened when discrepancies were observed in the methods (lines 148-151):**

| **Original text** | **Revised text** |
| --- | --- |
| Consensus was reached for any discrepancies in the data extracted between reviewers. | **Discrepancies in data were discussed between reviewers and the most appropriate data to include were determined through consensus. Consensus was achieved for all discrepancies.** |

7. Methods (search strategy and inclusion): The Recruitment Technique does not seem clear to me, please clarify.

**Re: With all due respect, we are uncertain as to what this comment entails. There was no recruitment for this study, as it was a meta-analysis. We also included the search terms (please refer to S1 Table) to recruit studies, as it were.**

8. Methods (search strategy and inclusion): Why 3 years (60 months) what are the studies or scientific documents that give the limit of 3 months to 60 months, because PrEP must avoid the risk of contamination so it must be continuous, right?

**Re: To be clear, 60 months is technically 5 years. Prior to conducting the meta-analysis, it was clear that no study had data beyond 60 months (as we were doing extensive literature searches for other studies). There is no hard documentation that it must be taken for up to 5 years.**

**In fact, PrEP only needs to be taken when an individual engages in an activity that is associated with HIV acquisition. This does not necessarily have to be continuous, hence why we included studies involving both daily and event-driven PrEP.**

9. Methods: In this methodological part, as you have used PRISMA, please present clearly: PICOS

P= Populations, I= Interventions, C=comparator, O= outcome, S= Study

**Re: We have now clearly mentioned each of these aspects in the section on inclusion criteria (lines 108-126):**

| **Original text** | **Revised text** |
| --- | --- |
| We included only studies that reported on retention of people using oral PrEP for HIV (i.e., fixed dose combination of emtricitabine and tenofovir disoproxil fumarate) taken daily, event-driven or both, and that were conducted among a predominantly (≥60%) MSM and/or TGP population. Moreover, studies were included if follow-up time and proportion of participants retained in care from PrEP initiation to discontinuation were reported or could be calculated for at least one of the pre-defined follow-up time points (i.e., beginning at month 3 since PrEP initiation, and continuing for every 3 months until month 60). Eligible study designs were: unblinded randomized controlled trials (RCT), open label extension (OLE) studies, longitudinal observational cohort studies, demonstration projects, implementation projects, and studies conducted in primary care or other routine public health settings. | We included studies **conducted in predominantly MSM and/or TGP populations (i.e., ≥60% of the study population consisted of these key populations) who were using oral PrEP for HIV** (i.e., fixed dose combination of emtricitabine and tenofovir disoproxil fumarate) taken daily, event-driven or both. **No comparison between regimens was required for inclusion.** **Studies were required to provide a quantitative measure of retention on PrEP.** **We categorized definitions of retention related to the following: PrEP continuation, where PrEP was still being used by the participant according to self-reports, prescription data, etc.; follow-up, where participants were still actively in care or enrolled in a study; mixed/other, where composite definitions or a definition other than continuation or follow-up were used.** Studies were **therefore** included if follow-up time and proportion of participants retained **from PrEP initiation to end of follow-up** were reported or could be calculated for at least one of the pre-defined follow-up time points (i.e., beginning at month 3 since PrEP initiation, and continuing for every 3 months until month 60). Eligible study designs were: unblinded randomized controlled trials (RCT), open label extension (OLE) studies, longitudinal observational cohort studies, demonstration projects, implementation projects, and studies conducted in primary care or other routine public health settings. |

10. Methods (data extraction): How is it possible to do a follow-up or retention study with on-demand or event-related PrEP?

**Re: Good point. Regardless of PrEP modality (continuous or event-related), individuals on PrEP need to be regularly screened for STIs and ensure that they did not acquire HIV infection. PrEP retention here would be defined as an individual who no longer continues regular screening visits.**

**We need to point out that different definitions of retention were used across studies. (This issue is why we decided to include it as a covariate in the meta-analysis.) Some studies did use stopping PrEP pills as a definition of retention, which would only be applicable to continuous PrEP.**

**We have made it clearer why definitions are important in the introduction (lines 70-76):**

| **Original text** | **Revised text** |
| --- | --- |
| Retention outcomes may also be attributable to factors related to location, financing and implementation or research in PrEP care[9-11]. So far, no previous review study has provided an aggregated analysis on the extent to which such program-specific characteristics affect PrEP retention outcomes. | Retention outcomes may also be attributable to factors related to location, financing and implementation or research in PrEP care[9-11]. **Retention outcomes could also vary with the type of oral PrEP being used (i.e., daily or event/driven), while certain definitions of retention, such as PrEP intake or PrEP prescription, no longer apply to those using event-driven PrEP.** So far, no previous review study has provided an aggregated analysis of the extent to which such program-specific characteristics, **including specific definitions of retention**, affect PrEP retention outcomes. |

**We have also provided more information on what the terms for continuation represented (lines 115-119):**

| **Original text** | **Revised text** |
| --- | --- |
| **--** | **We categorized definitions of retention related to the following: PrEP continuation, where PrEP was still being used by the participant according to self-reports, prescription data, etc.; follow-up, where participants were still actively in care or enrolled in a study; mixed/other, where composite definitions or a definition other than continuation or follow-up were used.** |

**It should be noted that we had separate definitions labelled “retention” and “follow-up”. These are nearly identical and given the broad use of retention in the manuscript, we have labeled this as “follow-up”.**

11. Results (characteristics of included studies): For RCTs, how was the analysis performed if the arm does not contain the drug, is it ethical please?

**Re: For RCT without PrEP, these arms were not included in the analysis. This applied to very few studies in our analysis. We made this clearer in the methods section (lines 132-134):**

| **Original text** | **Revised text** |
| --- | --- |
| For open-label RCTs containing both an “intervention” and “standard of care” arm, each arm was included as a separate study cohort. | For open-label RCTs containing both an “intervention” and “standard of care” arm, each arm was included as a separate study cohort **if the arm included individuals using PrEP**. **If the arm did not include individuals using PrEP, it was not included in the study.** |

**These RCT were conducted at a time when the efficacy of PrEP was completely unknown, hence they were ethical at the time of completion.**

**Of note, the included RCTs did not pertain to groups using versus not using PrEP. The RCTs commonly compared other interventions, such as apps/consulting etc., in addition to PrEP care versus PrEP care alone (stated as “standard of care” in the table summarizing studies).**

12. Discussion: the problem posed by the discussion is that of the completeness of the studies and consequently of the extrapolation of the results: the majority of the studies are from North America, just 5% for each of the other parts of the world, so the discussion of this representativeness bias needs to be strengthened.

**Re: We fully agree. The following sentence has been added to the discussion (lines 499-502):**

| **Original text** | **Revised text** |
| --- | --- |
| **--** | **“Fifth, the vast majority of published studies represented European, Australian and North American settings. Although some studies from South East Asia, Africa and South America were included, the pooled estimates might not be fully representative of these and other non-included regions.”** |

13. Conclusion: renforcer la conclusion par les facteurs du programme qui influencent réellement la rétention des cibles sous la PrEP

**Re: Merci pour ce commentaire. Nous avons ajouté une phrase sur les facteurs qui pourraient influencer la rétention à la conclusion (lignes 527-530):**

| **Original text** | **Revised text** |
| --- | --- |
| **--** | **PrEP programs with more intense monitoring schedules or requiring that patients pay for some of their PrEP/STI testing services may face lower rates of retention; however, clearer evidence that these program factors do indeed have an effect on retention is needed.** |

**Bien entendu, il nous faut des autres études au long terme afin de confirmer ces résultats et il est fort probable que le contexte a un impact majeur sur les facteurs programmatiques.**
