# Supplementary material for: Oral pre-exposure prophylaxis retention among men who have sex with men and transgender persons: Systematic review and meta-analysis
Source: PLoS One. 2025 Oct 17;20(10):e0333494. doi: 10.1371/journal.pone.0333494 (PMC12533894; doi:10.1371/journal.pone.0333494)
Supplement: Attachment — Submitted filename: prep_discont_response_R2.docx [file pone.0333494.s013.docx]

**Reviewer #1**

This is a mostly well written paper which analyses retention in oral PrEP through systematic review and modeling.

**Re: We thank the reviewer for taking the time to carefully review our revised manuscript, especially given the many changes from the original version, and their helpful suggestions.**

I have the following suggestions:

- It would be helpful to explain a bit more about the definition of retention, and how this might differ for event driven and daily PrEP. For event driven PrEP- is taking PrEP for each high risk event considered "retained"? So if there were 3 high risk events in 3 months and PrEP was taken each time - is this retained?

**Re: We thank the reviewer for these questions.**

**As we have stated in the introduction, studying retention is difficult given the many different definitions used across studies. Retention has been defined using some aspect of follow-up (i.e. attending visits for PrEP dispensing, counselling or STI clinics), PrEP continuation (i.e. whether PrEP is still used, through self-report, pill counts, prescription data), or a mix of these approaches.**

**Because oral daily and event-driven PrEP require different dosing schedules, definitions of retention will have to reflect these differences. Both oral daily and event-driven PrEP entail some kind of follow-up in care or engagement in care (i.e., regular STI screening), hence defining retention by these criteria apply to either PrEP modality. If retention is based on PrEP continuation, defining retention for event-driven PrEP becomes more complex. In such cases, it would be important to consider PrEP coverage for events that are associated with HIV acquisition (or “high risk”). In the reviewer’s example, if an individual had 3 “high risk” events in 3 months and took PrEP correctly for each of these three events, this would be considered “continued on PrEP”, which was considered as “retained” in PrEP for most of the included studies. For clarity, we have expanded our explanation of retention in the introduction as follows:**

| **Original text** | **Revised text (lines 67-75)** |
| --- | --- |
| Retention outcomes could also vary with the type of oral PrEP being used (i.e., daily or event/driven), ~~while certain definitions of retention, such as PrEP intake or PrEP prescription, no longer apply to those using event-driven PrEP.~~ | Retention outcomes could also vary **based on different definitions of retention (e.g., PrEP continuation, follow-up in care), and these definitions will have to reflect the** type of oral PrEP being used (i.e., daily or event/driven). **For example, if retention is defined by follow-up in care (i.e., still receiving STI testing, PrEP refills or new prescriptions), this criterion could apply to both oral daily and event-driven PrEP. However, for an individual who uses event-driven PrEP and may discontinue PrEP because they are no longer engaging in activities associated with HIV acquisition, a definition based on PrEP continuation would need to consider this risk and eventually whether the person is still engaged in care.** |

**We do recognize the issue of definition variability and it could be that these different definitions are lending to more variation across studies. To capture this, we recorded how each study defined retention under the variable “retention methodology”. The majority of studies (63%) used follow-up as their primary measure of retention. Our meta-regression analysis did not find an association between retention methodology and retention rates (please refer to Table 2 under “Outcome definition”).**

- It is not clear why TGP and MSM only were included as there are other groups at risk of HIV not included. This could be better justified in the intro.

**Re:** **Indeed, there are other groups who are (behaviorally) vulnerable to HIV, including people who inject drugs, adolescent girls and young women, and heterosexual individuals, also depending on the region. When we first considered the eligibility criteria for this meta-analysis, we noticed that there were very few studies conducted in these populations and most of the narrative on PrEP use highlighted the unique barriers and facilitators to PrEP retention for these key populations, including stigma, healthcare access, adherence perceptions and structural barriers (Koechlin FM et al,**

**AIDS Behav. 2017 May;21(5):1325-1335). Amid this substantial variation, this would only increase heterogeneity and potentially make these results less informative. For these reasons, we decided to focus on key populations for which more extensive data were available.**

**We have made this more explicit in the methods section:**

| **Original text** | **Revised text (lines 112-114)** |
| --- | --- |
| **--** | **We did not include studies exclusively without MSM or TGP because the barriers and facilitators to PrEP retention for these other key populations are likely different (14) and could increase heterogeneity.** |

**We have also urged future research to include more studies in these other populations in the Discussion:**

| **Original text** | **Revised text (lines 400-402)** |
| --- | --- |
| **--** | **Further meta-analyses should strive to include studies in key populations other than MSM and TGP as more data become available.** |

- Line 107 - 114 in methods - is a little unclear to me. Not sure it follows at line 111 where is says "studies were THEREFORE included....." Maybe delete the therefore since it does not seem to follow. I am also unclear what the definition of retention is as it is not actually stated anywhere - categorizations of retention are described but it is confusing then to include "mixed/other, where composite definitions or a definition other than continuation or follow-up were used."

**Re: Agreed. The “therefore” has been deleted. We have attempted to more thoroughly clarify the definitions of retention in the manuscript. The last bit on mixed/other is self-explanatory and hence we deleted the confusing text. The following modifications were made:**

| **Original text** | **Revised text (lines 116-120)** |
| --- | --- |
| We categorized definitions of retention related to the following: PrEP continuation, where PrEP was still being used by the participant according to self-reports, prescription data, etc.; follow-up, where participants were still actively in care or enrolled in a study; mixed/other~~, where composite definitions or a definition other than continuation or follow-up were used~~. | We categorized definitions of retention related to the following: PrEP continuation, **where retention was defined by whether someone was still on PrEP** according to self-reports, prescription data, etc.; follow-up, where **retention was defined by whether participants** were still actively in care or enrolled in a study; mixed/other. |

- Line 116 - not clear what studies conducted in primary care/public health settings means in this context. Suggest sticking to study designs in this section.

**Re: Our apologies for the confusion. We meant that we also considered analyses of routine care data for inclusion. This statement has now been revised for clarity.**

| **Original text** | **Revised text (lines 124-128)** |
| --- | --- |
| Eligible study designs were unblinded randomized controlled trials (RCT), open label extension (OLE) studies, longitudinal observational cohort studies, demonstration projects, implementation projects, and studies conducted in primary care or other routine public health settings. | Eligible study designs were unblinded randomized controlled trials (RCT), open label extension (OLE) studies, longitudinal observational cohort studies, demonstration projects, implementation projects, and studies **that analyzed routine care data from** primary care or other public health settings. |

- Line 117 - it seems odd to me to exclude blinded RCTs. Can you give a reference to support the statement that excluding RCTS means that outcomes approximate real world settings?

**Re: We appreciate the reviewer’s comment. The decision to exclude double- and single-blinded RCTs was based on the understanding that the unblinded trials and observational studies more appropriately approximate real-life conditions. These studies typically have broader inclusion criteria, with participants being more representative of real-world populations, and take place in less controlled settings, allowing participants to behave as they would in everyday life. Specifically, in the context of PrEP, participants’ awareness of whether they are receiving an effective HIV prevention strategy could influence their behavior (Underhill K. N Engl J Med. 2011;364(14):1374; author reply).**

**We have clarified that our intent was not to imply that excluding blinded RCTs “ensures” real-world settings, but rather to approximate them as closely as possible:**

| **Original text** | **Revised text (lines 128-129)** |
| --- | --- |
| Double- and single-blinded RCTs were excluded to ensure that the outcomes approximated real-world settings. | **Double- and single-blinded RCTs were excluded to more appropriately approximate real-world settings (15).** |

- Line 145-149 methods - it would be good to include a variable which allows us to understand whether these studies are from high income countries? I assume most are.

**Re: Agreed. This variable has been added as part of the meta-analysis:**

| **Original text** | **Revised text (lines 153-155)** |
| --- | --- |
| Data were extract on the following: region (i.e., North America, Europe, Australia, South America, Africa, Asia, or multiple); …. | Data were extract on the following: region (i.e., North America, Europe, Australia, South America, Africa, Asia, or multiple); **country income from the World Bank (i.e., regions were classified as low to middle income, high income, or multiple income levels);…** |

**With presentation of results:**

| **Original text** | **Revised text (lines 322-323)** |
| --- | --- |
| In contrast, studies conducted in Africa (vs. North America; p=0.005), **…** | In contrast, studies conducted in Africa (vs. North America; p=0.005), **studies from LMIC (vs. HIC, p=0.035),…** |

**And considered in the discussion:**

| **Original text** | **Revised text (lines 412-415)** |
| --- | --- |
| Furthermore, there were regional differences in loss of PrEP retention rates. When compared to North America, studies in Europe and Australia had lower loss of retention rates, while those in Africa had higher rates. | Furthermore, there were regional differences in loss of PrEP retention rates. When compared to North America, studies in Europe and Australia had lower loss of retention rates, while those in Africa had higher rates. **When dividing these countries based on income, loss of PrEP retention was more commonly observed in LMIC settings.** |

- Line 306 - I find "lower loss of PrEP retention rates" hard to understand. Can this be reworded?

**Re: Our apologies for the confusing phrasing. We are modeling the loss of PrEP retention, which is a term difficult to combine with the qualifiers “lower” and “higher”. We have reworded it as follows:**

| **Original text** | **Revised text (lines 321-322)** |
| --- | --- |
| … significantly lower loss of PrEP retention rates …. | … loss of PrEP retention rates **that were significantly lower (i.e., increased retention)**. |

- Please include more references in the discussion to support statements. For example in the discussion the authors report that there is poorer retention with more frequent clinic visits - have others also found this? Or is this a novel finding?

**Re: We have now carefully reviewed the discussion and have added several references to support our claims. If there are any remaining claims without sufficient reference, please let us know.**

**With regard to the comment on more frequent visits – this result is somewhat puzzling and as described in the discussion, this could have been the result of other setting contexts. There have been few studies that have examined this issue, yet we added some discussion:**

| **Original text** | **Revised text (lines 431-436)** |
| --- | --- |
| Loss of PrEP retention rates were found to be significantly lower among studies with ≥3 monthly follow-up visits (vs. <3 monthly), suggesting that those who are required to engage in more intense PrEP care are less likely to be retained on PrEP. The experienced burden of frequent clinic visits (11) or changing sexual behavior over time are likely reasons for this finding. | Loss of PrEP retention rates were found to be significantly lower among studies with ≥3 monthly follow-up visits (vs. <3 monthly), suggesting that those who are required to engage in more intense PrEP care are less likely to be retained on PrEP. The experienced burden of frequent clinic visits (11) or changing sexual behavior over time are likely reasons for this finding. **The effect of increased monitoring on PrEP adherence has rarely been quantitatively studied, but has surfaced as a prominent issue in several qualitative studies (38,39).** |

- Please discuss more the implication of there being only 1 study of only event driven PrEP and also that 40% of studies did not specify whether it was event driven or daily PrEP.

**Re: Thank you for the suggestion. We have added the following to the discussion:**

| **Original text** | **Revised text (lines 448-451)** |
| --- | --- |
| -- | **Only one study involved solely event-driven PrEP, which makes inference on this PrEP modality challenging. Roughly 40% of the included studies did not report PrEP modality and if these studies did include those undergoing event-driven PrEP, it would have helped provide a clearer understanding of PrEP retention for these users.** |
